# Constraints on lateral gene transfer in promoting fimbrial usher protein diversity and function

**DOI:** 10.1098/rsob.170144

**Published:** 2017-11-15

**Authors:** Christopher J. Stubenrauch, Gordon Dougan, Trevor Lithgow, Eva Heinz

**Affiliations:** 1 Infection and Immunity Program, Department of Microbiology, Monash University, Clayton 3800, Australia; 2 Infection Genomics Program, Wellcome Trust Sanger Institute, Hinxton CB10 1SA, UK

**Keywords:** outer membrane, translocation and assembly module, fimbriae

## Abstract

Fimbriae are long, adhesive structures widespread throughout members of the family Enterobacteriaceae. They are multimeric extrusions, which are moved out of the bacterial cell through an integral outer membrane protein called usher. The complex folding mechanics of the usher protein were recently revealed to be catalysed by the membrane-embedded translocation and assembly module (TAM). Here, we examine the diversity of usher proteins across a wide range of extraintestinal (ExPEC) and enteropathogenic (EPEC) *Escherichia coli*, and further focus on a so far undescribed chaperone–usher system, with this usher referred to as UshC. The fimbrial system containing UshC is distributed across a discrete set of EPEC types, including model strains like E2348/67, as well as ExPEC ST131, currently the most prominent multi-drug-resistant uropathogenic *E. coli* strain worldwide. Deletion of the TAM from a naive strain of *E. coli* results in a drastic time delay in folding of UshC, which can be observed for a protein from EPEC as well as for two introduced proteins from related organisms, *Yersinia* and *Enterobacter*. We suggest that this models why the TAM machinery is essential for efficient folding of proteins acquired via lateral gene transfer.

## 1. Introduction

Bacteria can acquire new phenotypes to adapt to changing environments through mutations of their genome and through the acquisition of new genes. Genes acquired through lateral gene transfer (LGT) are particularly important for the adaptation of bacterial pathogens, providing them with means to invade and conquer new niches and often to promote their virulence [1–7]. In many cases, the selectable phenotypes arising from the LGT are due to a monomeric enzyme or pump that promotes resistance to a heavy metal or antimicrobial compound. However, some phenotypes require multimeric structures, encoded on multiple genes and ultimately assembled by the host cell's assembly machinery. The success or failure to assemble complicated cellular machinery acquired through LGT would be a key hurdle in the evolutionary success of bacterial lineages adapting to changing environments. Stated simply, acquiring genes that encode a virulence factor ultimately needs to be followed by the assembly of a functional form of the virulence trait in order to effect a phenotypic outcome.

Efficient attachment to host cells is one of the key virulence factors essential for many bacterial pathogens. The 2011 outbreak of *Escherichia coli* STEAEC O104:H4, which was hallmarked by a high morbidity and mortality rate, is understood as a new configuration of known virulence factors: a combined effect on better adherence through an acquired adhesion system (Iha), which in turn provided for better delivery of the Stx toxin to host cells with devastating effect [8–10]. This is an example of how crucially important pathogen–host cell adhesion is to successfully establish infection in the human host for specific *E. coli* pathotypes. Among the arsenal of adhesive structures in Gram-negative bacteria, the most important are fimbriae or pili, which are multimeric, extracellular fibres. In addition to the multiple subunits that form each fimbrial fibre, a set of membrane-embedded and periplasmic proteins form the molecular machinery to extrude the fimbriae across the bacterial outer membrane. Key examples of these molecular machines have been the subject of an impressive array of structural and functional studies [11,12], and genome sequencing studies are identifying a growing number of further, uncharacterized systems. The chaperone–usher systems are usually encoded in operons of genes, and comprise at least four subunits: a chaperone to aid assembly and transport of the fimbrial subunits within the periplasm; in most cases at least two types of fimbrial subunits, including a tip adhesin that confers binding specificity and the major fimbrial subunit that comprises the bulk of the structure; and an usher protein, which serves as the membrane conduit through which the fimbriae are translocated [13]. Their classification system is based on Greek letters (alpha-, beta-, gamma-, etc.) with the usher protein used as the basis of the classification [13]. While the fimbrial subunits require their cognate chaperone and usher for assembly [12], recent work suggests that, in turn, the usher proteins—which are beta-barrel outer membrane proteins—require the beta-barrel assembly machinery (BAM) complex and the translocation and assembly module (TAM) in order to be effectively assembled into the bacterial outer membrane [14].

The BAM complex is essential for the assembly of outer membrane proteins, and the core gene *bamA* is essential for bacterial cell viability [15–17]. The TAM is widely distributed across Gammaproteobacteria [18,19], and is involved in the biogenesis of outer membrane proteins such as autotransporter adhesins [20], inverse autotransporter adhesins [21] and fimbrial ushers [14]. However, the TAM, consisting of the protein subunits TamA and TamB [20], is not essential for cell viability. It has therefore been unclear what selective pressure is in place to have *tamA* and *tamB* maintained across the Gammaproteobacteria. The current hypothesis is that the TAM assists in the folding and assembly of proteins that have complex structures [22]. In principle, this may include alien proteins acquired from other bacteria via LGT.

Here, we assess the diversity of fimbrial usher proteins across an extensive collection of enteropathogenic *E. coli* (EPEC) and extraintestinal pathogenic *E. coli* (ExPEC), especially uropathogenic *E. coli* (UPEC) and subsets of other species from the Enterobacteriaceae. Analysis of this large collection emphasized earlier observations that the usher proteins have a non-uniform presence in *E. coli* [23]. We find that some usher proteins are highly conserved, suggesting that, for a significant time period, they have served core functions in *E. coli*. Other ushers are much more distinctly distributed, which suggests a more recent acquisition and/or a more specialized function. The complex distribution is further reflected more broadly across the Enterobacteriaceae. LGT is the most likely method of dissemination, given the highly uneven distribution not only in *E. coli* but also when considering other genera such as *Salmonella*, *Enterobacter*, *Yersinia* and *Klebsiella*. We show that the TAM machinery is important in the folding of an alien sequence from *Enterobacter asburiae* and *Yersinia enterocolitica* into the outer membrane of *E. coli*, and suggest that selective pressures favouring exchange of large surface proteins through LGT contribute to the maintenance of cellular factors such as the TAM. Large adhesins and other unusual outer membrane-embedded cell surface proteins are frequently exchanged on mobile elements and thus acquired through LGT to new host cells. Having a folding machinery that significantly contributes to the speed with which these large structures can be used for phenotypic advantages is crucial for the invasion of some niches, such as the urogenital tract [14]. We followed on this observation, and investigated the evolutionary history of the usher family, which form the basis of fimbriae, one of the best-studied group of adhesive structures, and the folding kinetics of several usher proteins in a heterogeneous host.

## 2. Results

While *E. coli* can be considered a model organism and is a commensal of humans, it is also a significant human pathogen. Pathotypes of *E. coli* have been shown to employ a diverse range of fimbriae to ensure infection [13,23,24], which translates to the variability in adherence to different host cells among EPEC and ExPEC (especially UPEC) strains. Although genomic insights over the recent years have blurred the lines between the different pathotypes [25], there has been a recent expansion of knowledge about the diversity of *E. coli*, including our understanding of the paraphyletic origins of EPEC [26–28]. We therefore sought to assess whether (and how) the diversity of fimbriae–usher adhesive systems is reflected in *E. coli* pathotypes.

**Figure 1.**
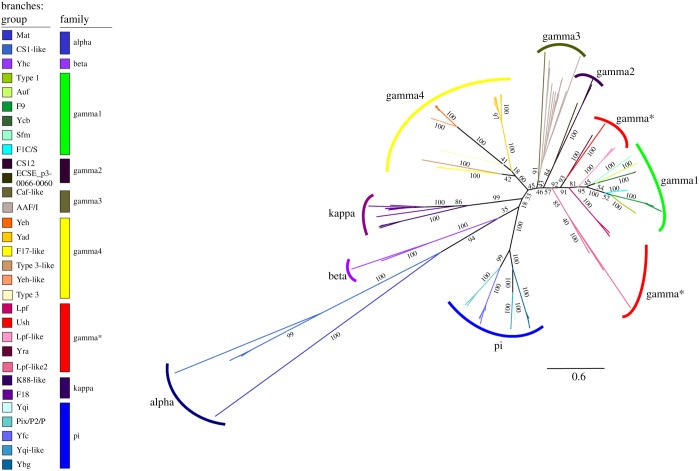
Phylogeny of usher proteins in a large *E. coli* collection. The *E. coli* genomes (electronic supplementary material, table S3) were searched using HMMER and the Pfam profile for usher proteins and subjected to tree calculation using RAxML. Following manual assessment visually, monophyletic groups are coloured according to their described members (electronic supplementary material, table S4); four groups without described members as in Wurpel *et al.* [23] are based on the annotation of similar sequences in UniProt (LPF-like 2, AggC (AAF/I), FedC (F18), MrkH (Type 3)).

**Figure 2.**
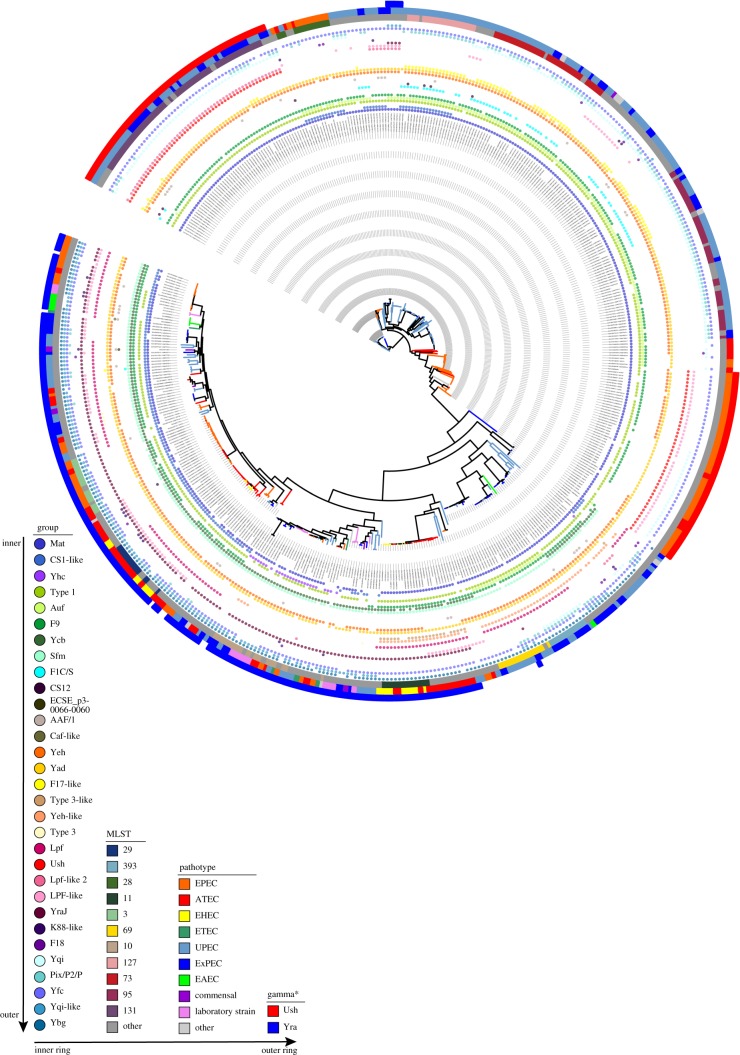
The distribution of ushers across the *E. coli* pangenome. Given the recent increase in publicly available EPEC/UPEC genomes [26–29], we investigated the distribution of ushers across *E. coli*. The tree is based on a core gene alignment of *E. coli* genomes with a focus on EPEC and ExPEC strains, but also including a variety of reference strains for other pathovars. The inner rings show the respective usher families, the other rings show, from inside to outside, the main sequence types according to multi-locus sequence typing (MLST), and the pathotypes. The presence of UshC and YraJ are again highlighted in the outermost ring. This highlights the uneven distribution of the two closely related usher proteins UshC and YraJ, both across the *E. coli* diversity but also within the respective pathovars; branches are coloured according to the pathovars scheme as indicated in the legend. The tree representation was performed using iTOL [30]. Pathotypes: EPEC, enteropathogenic *E. coli*; ATEC, atypical EPEC; EHEC, enterohaemorrhagic *E. coli*; ETEC, enterotoxigenic *E. coli*; UPEC, uropathogenic *E. coli*; ExPEC, extraintestinal pathogenic *E. coli*; EAEC, enteroaggregative *E. coli*; other, see details in electronic supplementary material, table S3.

A broad database of a selection of *E. coli* genomes ([26–29]; electronic supplementary material, table S3) was built and analysed by hidden Markov Model search using HMMER to identify all encoded usher proteins in the dataset. Initially, to classify sequences without functional annotations, we spiked our dataset with annotated sequences [23], and after removing highly similar sequences, we defined usher groups based on manual assessment of monophyletic branching with reference sequences (figure 1). This revealed several branches in the tree that had no clear association with previously described families or represented divergent branches (e.g. ‘Lpf-like 2’ is most similar to proteins annotated Lpf-like in UniProt, but clearly distinct from the described Lpf-like), indicating that we are only beginning to appreciate the diversity to be found in *E. coli* usher sequences. Based on this classification, we incorporated the monophyletic groups into our full dataset, to investigate the distribution of usher proteins across this large dataset (figure 2).

This analysis revealed a peculiar distribution of members of a Gamma subfamily, designated Gamma* (‘Gamma star’, in accordance with [23]). Within this subfamily, we identified an *E. coli* protein which we refer to as UshC (locus tag identifer ECSF_0165), which is located in an operon consisting of the fimbrial subunit *ushA*, chaperone *ushB*, usher *ushC* and fimbrial subunit *ushD* (electronic supplementary material, figure S4). UshC showed a distinct distribution in ExPEC and EPEC lineages and is found in the globally distributed and most abundant circulating multi-drug-resistant ExPEC sequence type ST131 [31,32]. Especially within the EPEC lineages [26–28], we observe a distribution and maintenance in a large group of strains containing the model strain O127:H6 E2348/69, as well as in the interspersed isolates of other pathotypes. The distribution of UshC across the tree also strongly suggests distribution via lateral transfer, given the distant relation between the EPEC groups and ST131 (figure 2). UshC, furthermore, shows an inverse correlation with another member of this family, YraJ (figures 1 and 2), with which it is closely related and which was found in almost all other EPEC strains (figure 2).

**Figure 3.**
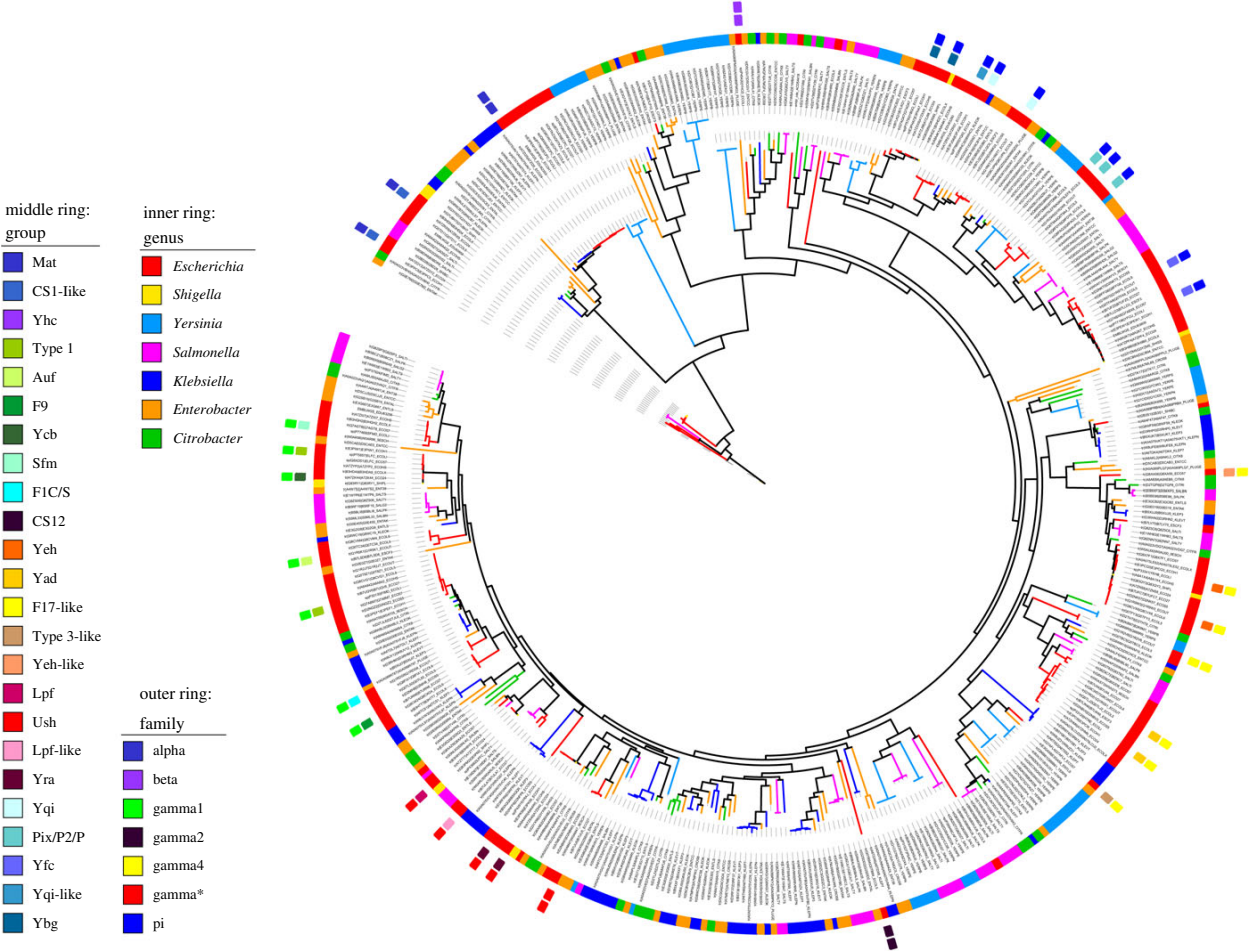
The phylogenetic relationships of usher proteins in Enterobacteriaceae reference strains. The tree was generated using RAxML and shows the diversity of usher proteins across several genera as given in the electronic supplementary material, table S1 and highlights that these proteins are very widely distributed across various species within the Enterobacteriaceae. The colours indicate the different genera (inset). Chaperone–usher systems are assigned as in figure 1, but only for the representative *E. coli* proteins as shown in the middle and outer ring fragments.

To gain further insights into how representative the distribution is for other bacterial species, we investigated the diversity of ushers across model organisms within the Enterobacteriaceae (figure 3). This analysis emphasized that usher proteins are widely distributed, and that the different families previously based on *E. coli* representatives [13,23] can be found in a variety of organisms. However, usher proteins are not evenly distributed within the different genera. When considering selected representative species, it is clear that often unrelated genera are more similar with respect to usher families than species within the same genus (figure 3). This is in accordance with the typical distribution of adhesins [21] and other virulence factors, highlighting the importance of LGT for the dissemination of fimbriae [33].

**Figure 4.**
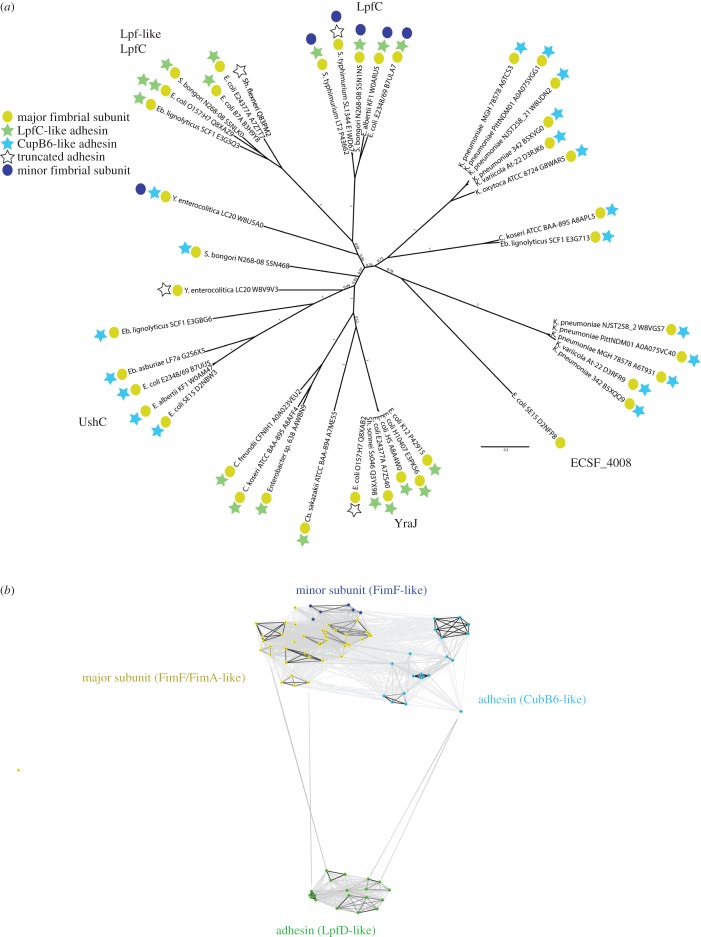
The Ush/Yra clade ushers. (*a*) Phylogenetic tree of the respective usher sequences calculated with MrBayes shows the various adhesins associated with the respective usher sequence. The nonlinear evolution of the chaperone–usher systems is apparent from the different monophyletic groups displaying a mixed distribution of associated adhesin sequences in the operons. (*b*) Similarity network of the sequences as in the electronic supplementary material, table S5, highlights two different types of adhesins associated with the different operons; one group comprises stalk-like adhesins, which can also form the tip, whereas the other group includes a second adhesion protein different to the stalk-like sequences.

Further analysis of the Gamma* subfamily of chaperone–usher systems closely related to UshC and YraJ revealed several monophyletic lineages for the usher proteins (figure 4*a*), emphasizing their independent acquisition in the different *E. coli* lineages (figure 1). The current model for creating diversity in the chaperone–usher systems available to a species posits that the entire chaperone–usher operon is mobile during LGT. Phyre2, a sequence/structure comparison tool [34], revealed that there are distinct adhesins present in the UshC and YraJ subgroups. While some are more similar to the LpfD adhesin [35], others are more similar to the *Pseudomonas* adhesin CupB6 ([36]; figure 4*b*; electronic supplementary material, table S5). This dichotomy is further reflected in the chaperones that assemble the adhesin subunits (electronic supplementary material, figure S2), which cluster similarly to the phylogenetic history of the usher proteins, largely consistent with an LGT event acting upon the entire chaperone–usher operon, although the ordering of the inner branches is not stable with low support values. In addition, we see several cases of pseudogenization or loss of the chaperones as well as fimbrial adhesins (electronic supplementary material, table S2), which further highlights the dynamic nature of these operons also once they have been incorporated into the chromosome. The capacity for fimbriae to carry different tip structures is well studied and often used as a surface display system [37]. However, these experiments also only report on steady-state expression levels, and not how rapidly or efficiently expression is enacted.

**Figure 5.**
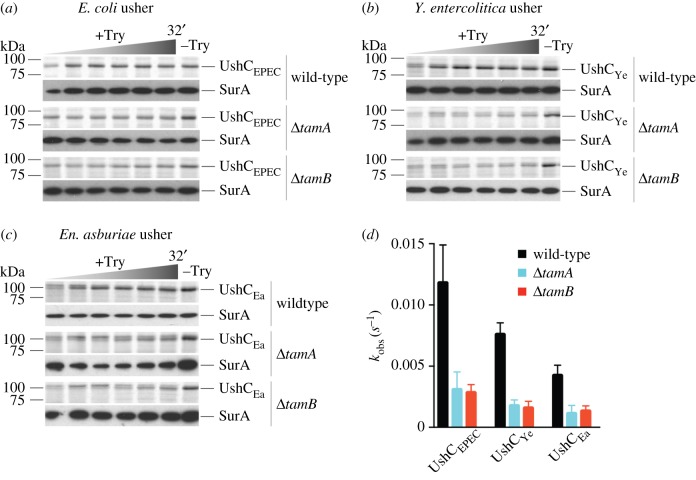
Usher biogenesis in *E. coli. Escherichia coli* cells harbouring (*a*) pCJS39, (*b*) pCJS75 or (*c*) pCJS77 were assessed by pulse chase analysis. Aliquots were taken at 10 s, 2, 4, 8, 16 and 32 min, treated with (+Try) or without (−Try, last timepoint only) 20 μg ml^−1^ trypsin. Analysis was by SDS–PAGE, storage phosphor-imaging and immunoblotting. Representative autoradiograms and immunoblots are shown, from three independent experiments (*n* = 3). The time increment is indicated as a graded triangle above the autoradiogram. SurA is a periplasmic protein used to assess the integrity of the outer membrane. (*d*) The usher densities at each timepoint (*a*–*c*) were used to calculate the observed rate constants (*k*_obs_). Calculations were as per Stubenrauch *et al.* [14]. Error bars represent s.e.m. (*n* = 3), and all folding rates of mutants were significantly slower than the respective wild-type folding rate, as assessed by one-way ANOVA (*p* < 0.05).

While the fimbrial subunits are folded with the chaperone encoded within the operon, the usher itself needs the cellular beta-barrel assembly machinery. To address the assembly mechanism for UshC_EPEC_ (the UshC from EPEC O127:H6 E2348/69; UniProt: B7UIJ5), a biochemical assay was established wherein UshC_EPEC_ was expressed under the control of a T7 RNA polymerase-driven promoter in *E. coli* BL21 Star™ (DE3). In this system, transcription by *E. coli* RNA polymerase is repressed, and ^35^S-labelled amino acids are incorporated into the protein of interest [14], allowing for its detection by radiography. Analysis of UshC_EPEC_ assembly revealed that, relative to the levels of usher assembly seen in wild-type *E. coli*, in the absence of either *tamA* or *tamB*, there was a decrease in the amount of functionally assembled usher (figure 5*a*; electronic supplementary material, figure S5).

The irregular distribution of fimbrial operons across the taxonomic range (figures 2 and 3) and the importance and apparent frequency of LGT leading to their distribution led us to test whether the *E. coli* host machinery would be able to assemble the products of newly acquired fimbrial ushers. Homologues UshC_Ye_ (from *Y. enterocolitica* LC20; UniProt W8V9V3) and UshC_Ea_ (from *En. asburiae* LF7a; UniProt G2S6X5) showed a significant decrease in the amount of functionally assembled usher (figure 5*b*,*c*; electronic supplementary material, figure S5). Densitometric analyses revealed the observed rate constant for the assembly of the protease-resistant (assembled) UshC was significantly greater when catalysed by the TAM (figure 5*d*), for the recently acquired UshC_EPEC_ and even more so for the alien sequences from *Y. enterocolitica* and *En. asburiae*.

## 3. Discussion

Genome plasticity particularly through LGT has been suggested as the key to the great variability seen in the various pathotypes of *E. coli*, by enabling constant alterations to the fitness and resultant competitiveness of individuals in specific niches [4]. Many studies in comparative genomics support this concept of *E. coli* genome plasticity; the core genome of *E. coli* K-12 substr. MG1655, EHEC O157:H7 and UPEC CFT073 encodes approximately 40% of their proteome [38]. The distribution of chaperone–usher systems contributes to this diversity in proteome and adaptive fitness in *E. coli* lineages.

Adhesion is an essential step for many human pathogens to anchor themselves in their respective niche. In the case of uropathogenic or enteric pathogens, failure to rapidly and effectively adhere impacts on colonization, given that flushing action of constant fluid movement is one of the main challenges facing bacteria in these environmental niches. Fimbriae play an essential role in the adhesion process, and a delay in their expression equates to a failure to adhere in host niches [14,39]. It is perhaps because of their importance in pathogenic lifestyles and host interactions that adhesins are (i) often shared via LGT [40] and (ii) undergo rounds of adaptation to enhance host interaction or evasion of the immune system through positive selection and/or recombination [41–43]. A high number of transposable elements associated with the usher operons were detected (electronic supplementary material, table S2), and several cases of potential pseudogenization were observed, mainly of the chaperone, through frameshifts. Fimbrial operons are a highly dynamic locus in most genomes, regarding both their occurrence/absence and precise sequence [41,42].

We analysed the diversity of fimbrial usher distribution in a large collection of *E. coli* whole-genome data with a focus on EPEC and ExPEC. In many cases, a large number of fimbrial loci could be encoded within a single strain, in some cases up to 16 (figure 2). Despite this, YraJ and UshC were never simultaneously encoded by any *E. coli* lineage (figure 2). Mutual exclusion has been observed among other classes of outer membrane proteins. It has been hypothesized that this occurs as result of environmental specificity, incompatibility or functional redundancy, or to avoid interference in similar target sites [44]. It is not clear, however, how any of these factors would impact to keep a mutual exclusivity between *yraJ* and *ushC*, especially if their target adhesins have the potential to have distinct specificities [41], and if this observation remains supported when further *E. coli* sequences keep being analysed.

**Figure 6.**
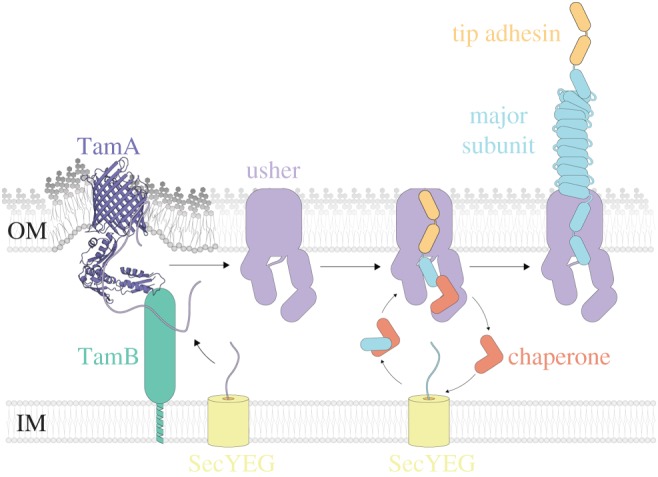
Schematic of fimbriae biogenesis. Nascent protein is translocated across the inner membrane (IM) via the SecYEG apparatus. The TAM is thought to promote protein insertion through destabilization of the lipid bilayer [50,51]. TamA (pdb: 4C00) acts as a lever, pushing onto TamB, to distort the outer membrane (OM). Once assembled, the fimbrial usher acts as an anchor and pore for fimbrial subunits to thread through. Initially, the dedicated chaperone transfers the tip adhesion subunit to initiate fimbrial biogenesis. The chaperone subsequently transfers hundreds to thousands of the major fimbrial subunits, allowing the growing pilus to extend from the cell surface [12].

Genes transferred by LGT pose a potential risk to the cell, and are often initially silenced by systems such as the histone-like nucleoid structuring protein (H-NS) [45]*.* One such risk is that differences in codon usage between species will impact on translation rates in cells that attempt to express alien proteins acquired through LGT [46,47]. An additional risk would be the inhibition of protein assembly rates in cells that express alien proteins acquired through LGT. Studies in *E. coli* show that in the absence of the TAM, reduced assembly rates occur for model proteins like FimD [14]. The expression of adhesins is tightly regulated including complex counteracting factors [48], and several *E. coli* fimbriae–usher operons are under the control of H-NS, further highlighting the need for tight regulation and their likely role in virulence [49]. It is now also clear that for UshC, whose evolutionary history clearly indicates LGT between various lineages, the folding efficiency expressed is rate-limited by the TAM. This indicates that adhesins acquired by LGT (e.g. from *Y. enterobacter* and *En. asburiae*) would be more rapidly deployed and expressed to promote new phenotypes, provided that the TAM is present (figure 6). We suggest that this is an important function of *tamA* and *tamB* in the Proteobacteria, where they are almost universally conserved, despite their non-essential nature [18,19]. This underscores that there is no apparent limitation to the sharing of fimbrial clusters. Given the wide distribution across Enterobacteriaceae shown here, there seems to be no strictly imposed restriction to the protein sequence of the usher. This parallels the diversity of other adhesins assembled by the TAM machinery such as autotransporters and inverse autotransporters [20,21,52]. Despite its molecular complexity, the chaperone–usher system is highly adaptable for mediating bacterial adhesion and readily shared across bacterial species. A better understanding of the binding properties of the different fimbrial adhesins, combined with high-resolution sequence analysis such as shown here, provides insight into host range and tissue tropisms species of Enterobacteriaceae and will shed further light on the highly complex evolution of uro- and enteric pathogens.

## 4. Material and methods

### 4.1. Sequence analyses of reference strains

The full proteomes for the respective reference strains (electronic supplementary material, table S1) were retrieved from the UniProt database ([53]; last accessed 7 May 2015). The HMMER profile for ushers (PF00577.15) was retrieved from the Pfam website [54], and HMMER [55] was used to run a search (HMMER v. 3.0; hmmsearch using the -max option with all else default) against the combined file of all reference strain protein sequences. To identify non-usher contaminants from divergent usher sequences, a protein–protein similarity network was used to extract the sequences of all usher proteins following manual inspection of the formed clusters (CLANS [56]; *p*-value cut-off 1 × 10^−5^; electronic supplementary material, figure S1). Sequences with less than 600 amino acids were furthermore removed to remove contaminants. One divergent *E. coli* sequence was missing from the current dataset and added manually (E3PPC5 [23]). The operons of the reference strains as shown in figure 4 and the electronic supplementary material, table S2 and figures S2 and S3 were retrieved manually from Ensembl bacteria [57], and adhesins were clustered using CLANS (*p*-value cut-off 1 × 10^−10^; figure 4) to identify different types of adhesins. Alignments were performed with mafft [58] using the -linsi option, and informative sites selected using trimal with the auto-1 setting [59]. Trees were calculated using RAxML [60], MrBayes [61] or PhyloBayes [62] as indicated in the respective figure legends. Calculations for RAxML were performed with the fast bootstrap setting, and the model was set to PROTGAMMALGF with 100 bootstrap replicates; MrBayes was run for 1 million generations under the mixed amino acids model, with a burnin of 25% for the consensus tree; and PhyloBayes was run using the C20 (electronic supplementary material, figure S2*c*) or C60 (electronic supplementary material, figure S3*b*) model, and convergence was assessed manually with the bpcomp and tracecomp commands as suggested by the authors, consensus trees were calculated with 25% burnin.

### 4.2. Sequence analyses of enteropathogenic *Escherichia coli* diversity

For the *E. coli* diversity investigation, nucleotide sequences were retrieved from GenBank (for accession numbers, see electronic supplementary material, table S2), and to limit differences in gene/start site calling due to the different publication times and annotation software used for the included genomes, the assemblies were all annotated using prokka [63]. The core gene alignment was generated with roary [64], informative sites were chosen using snp_sites with default settings [65] and the tree calculation was performed using RAxML with the reversible GTR model and 100 bootstrap replicates. To find and distinguish the different usher in the dataset, a HMMER (v. 3.1 [55]) search with the Pfam profile PF00577.15 as described above was performed. All resulting hits were combined, and sequences with less than 600 amino acids removed as fragments/incomplete sequences. The remaining sequences were clustered with uclust [66] using the usearch–cluster_fast command at a cut-off of id 0.99, and the resulting centroids were used for a tree calculation. To facilitate distinguishing the different usher proteins, the sequence set was furthermore spiked with reference sequences for the main *E. coli* usher groups as indicated in the electronic supplementary material, table S3. The sequences were then aligned using muscle [67], and informative sites were chosen with the tcs online server [68]. The resulting reduced alignment was used as input for a tree calculation using RAxML with the LG model and empirical frequencies and 100 bootstrap replicates. The resulting tree was used to identify the centroids branching monophyletic with the different spiked usher sequences, and the presence or absence of sequences in the respective clusters is indicated in figure 2.

### 4.3. Functional analyses

Pulse chase analyses were performed in triplicate as described previously [14] with several modifications. Briefly, *E. coli* BL21 Star™ (DE3) wild-type, Δ*tamA* or Δ*tamB* strains were incubated to mid-log phase in LB media (37°C, 200 r.p.m. (25 mm orbit)), then transferred to M9-S media [14]. Following a 30 min incubation (37°C, 200 r.p.m. (25 mm orbit)), cells were treated for 1 h with rifampicin (200 μg ml^−1^, 37°C, 400 r.p.m. (3 mm orbit)) and induced for 5 min with IPTG (0.2 mM, 30°C, static). Cells were then ‘pulse’-labelled for 45 s with EXPRE^35^S^35^S, [^35^S]-Protein Labelling Mix (30 μCi ml^−1^, 30°C, static), containing 73% [^35^S]-methionine and 22% [^35^S]-cysteine (NEG072, Perkin Elmer), and then immediately subjected to centrifugation (5 min, 3000*g*, 4°C) and resuspended in M9 + S media [14]. Cells were then ‘chased’ for up to 32 min (30°C, static) and aliquots were taken at appropriate timepoints. Aliquots were treated with the exogenous addition of trypsin (to 20 µg ml^−1^) for 10 min on ice, before the total protein content of the samples was TCA-precipitated. The TCA-precipitated pellets were washed with acetone and resuspended in SDS loading dye. Samples were incubated for 10 min at 100°C and analysed by 12% SDS–PAGE. After electrophoresis, proteins were transferred onto 0.45 µm nitrocellulose membranes. Radiation was captured overnight using a storage phosphor screen (GE Health Sciences) and detected using a Typhoon Trio (320 nm). Immunoblotting for the presence of the control protein SurA was performed as per Leyton *et al.* [69].

## Supplementary Material

Supplementary info

## Data accessibility

The alignment and tree files are available under https://figshare.com/s/20b09a45736b6188fc32. All sequences used were retrieved from public databases, accession numbers of all used sequences are available in the electronic supplementary material, tables.
