# Supplementary material for: Constraints on lateral gene transfer in promoting fimbrial usher protein diversity and function
Source: Open Biol. 2017 Nov 15;7(11):170144. doi: 10.1098/rsob.170144 (PMC5717340; doi:10.1098/rsob.170144)
Supplement: Supplementary info [file rsob170144supp1.pdf]

Supporting information for: Constraints on lateral gene transfer in promoting fimbrial usher protein diversity and function.

Christopher J Stubenrauch[1], Gordon Dougan[2], Trevor Lithgow[1*], Eva Heinz[2*]

*Correspondence:
Eva Heinz, Infection Genomics Program, Wellcome Trust Sanger Institute, Hinxton, CB10 1SA, UK
eva.heinz@sanger.ac.uk
Trevor Lithgow, Infection and Immunity, Monash University, Clayton, 3800, Australia
trevor.lithgow@monash.edu

1: Infection and Immunity Program, Department of Microbiology, Monash University, Clayton 3800, Australia
2: Infection Genomics Program, Wellcome Trust Sanger Institute, Hinxton, CB10 1SA, UK

Figure S1

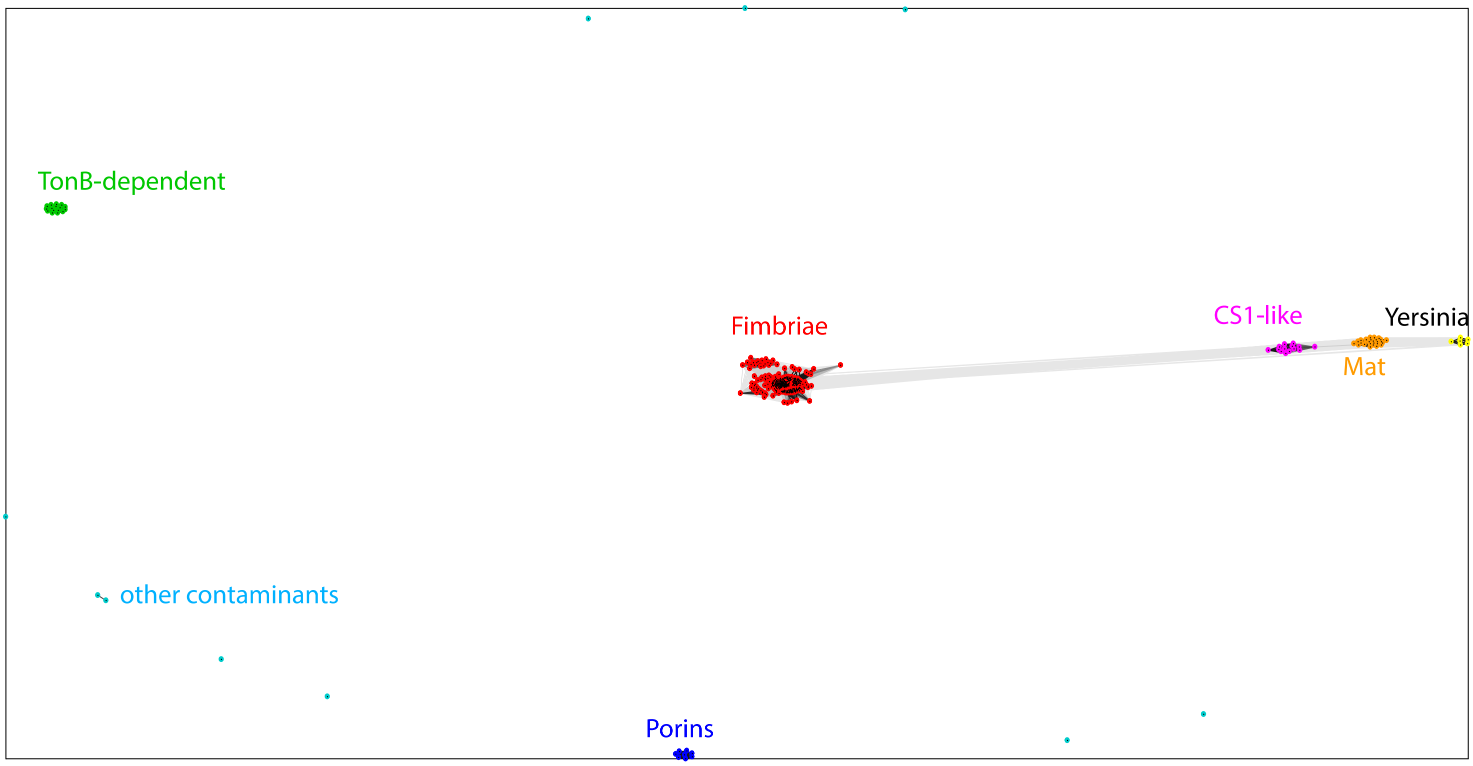
TonB-dependent
Fimbriae
CS1-like
Yersinia
Mat
other contaminants
Porins

Figure S1: The clustering of model organism usher search hits used to distinguish usher sequences from contaminants in the search results.

Figure S2

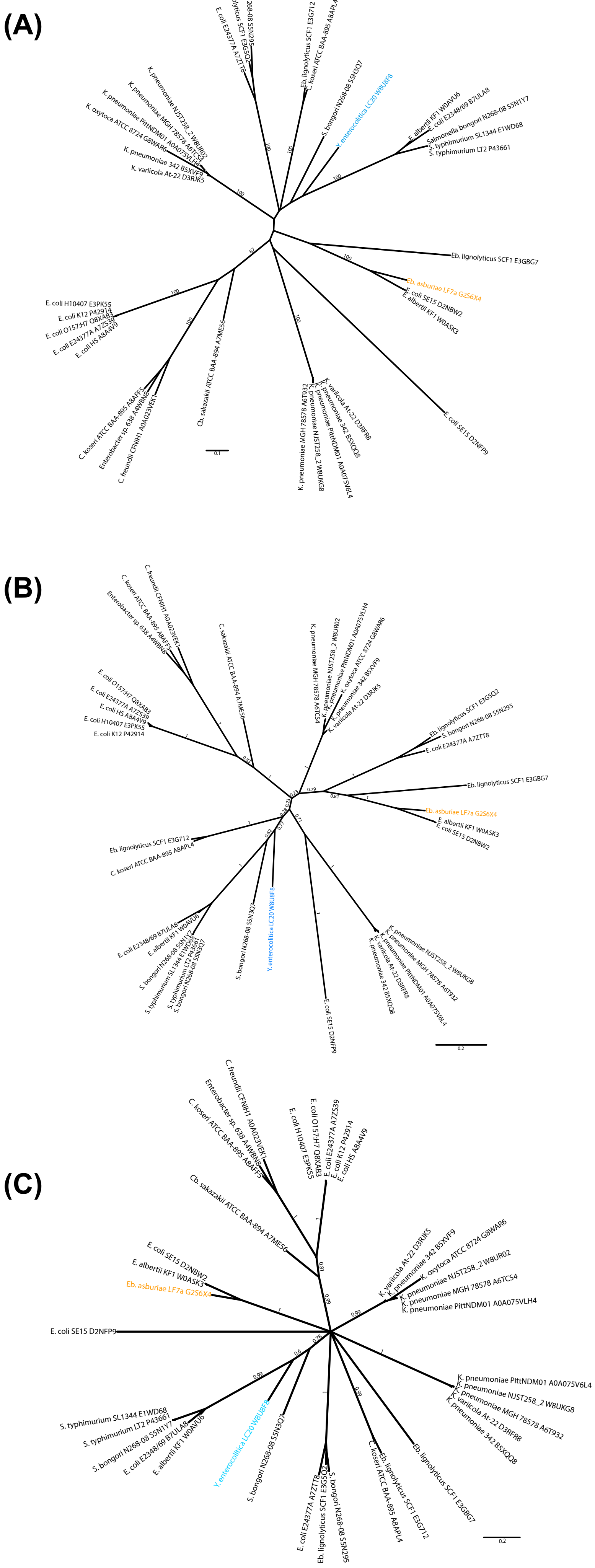
(A)
S. bongori N268-08 S5N295
Eb. lignolyticus SCF1 E3G5Q2
E. coli E24377A A7ZTT8
Eb. lignolyticus SCF1 E3G712
C. koseri ATCC BAA-895 A8APL4
S. bongori N268-08 S5N3Q7
Y. enterocolitica LC20 W8U8F8
E. albertii KF1 W0AVU6
E. coli E2348/69 B7ULA8
Salmonella bongori N268-08 S5N1Y7
S. typhimurium SL1344 E1WD68
S. typhimurium LT2 P43661
K. pneumoniae NJST258_2 W8UR02
K. pneumoniae MGH 78578 A6TCS4
K. pneumoniae PittNDM01 A0A075VLH4
K. oxytoca ATCC 8724 G8WAR6
K. pneumoniae 342 B5XVF9
K. variicola At-22 D3RJK5
Eb. lignolyticus SCF1 E3GBG7
Eb. asburiae LF7a G2S6X4
E. coli SE15 D2NBW2
E. albertii KF1 W0ASK3
E. coli H10407 E3PK55
E. coli K12 P42914
E. coli O157:H7 Q8XAB3
E. coli E24377A A7ZS39
E. coli HS A8A4V9
Cb. sakazakii ATCC BAA-894 A7ME56
C. koseri ATCC BAA-895 A8AFF5
Enterobacter sp. 638 A4WBN8
C. freundii CFNIH1 A0A023VEK1
K. pneumoniae MGH 78578 A6T932
K. pneumoniae NJST258_2 W8UKG8
K. pneumoniae PittNDM01 A0A075V6L4
K. pneumoniae 342 B5XQQ8
K. variicola At-22 D3RFR8
E. coli SE15 D2NFP9
0.1
(B)
C. freundii CFNIH1 A0A023VEK1
C. koseri ATCC BAA-895 A8AFF5
Enterobacter sp. 638 A4WBN8
C. sakazakii ATCC BAA-894 A7ME56
E. coli O157:H7 Q8XAB3
E. coli E24377A A7ZS39
E. coli HS A8A4V9
E. coli H10407 E3PK55
E. coli K12 P42914
K. pneumoniae MGH 78578 A6TCS4
K. pneumoniae NJST258_2 W8UR02
K. pneumoniae PittNDM01 A0A075VLH4
K. oxytoca ATCC 8724 G8WAR6
K. pneumoniae 342 B5XVF9
K. variicola At-22 D3RJK5
Eb. lignolyticus SCF1 E3G5Q2
S. bongori N268-08 S5N295
E. coli E24377A A7ZTT8
Eb. lignolyticus SCF1 E3GBG7
Eb. asburiae LF7a G2S6X4
E. albertii KF1 W0ASK3
E. coli SE15 D2NBW2
Eb. lignolyticus SCF1 E3G712
C. koseri ATCC BAA-895 A8APL4
E. coli E2348/69 B7ULA8
E. albertii KF1 W0AVU6
S. bongori N268-08 S5N1Y7
S. typhimurium SL1344 E1WD68
S. typhimurium LT2 P43661
S. bongori N268-08 S5N3Q7
Y. enterocolitica LC20 W8U8F8
E. coli SE15 D2NFP9
K. pneumoniae NJST258_2 W8UKG8
K. pneumoniae MGH 78578 A6T932
K. pneumoniae PittNDM01 A0A075V6L4
K. variicola At-22 D3RFR8
K. pneumoniae 342 B5XQQ8
0.2
(C)
C. freundii CFNIH1 A0A023VEK1
Enterobacter sp. 638 A4WBN8
C. koseri ATCC BAA-895 A8AFF5
Cb. sakazakii ATCC BAA-894 A7ME56
E. coli H10407 E3PK55
E. coli O157:H7 Q8XAB3
E. coli E24377A A7ZS39
E. coli K12 P42914
E. coli HS A8A4V9
E. coli SE15 D2NBW2
E. albertii KF1 W0ASK3
Eb. asburiae LF7a G2S6X4
E. coli SE15 D2NFP9
K. variicola At-22 D3RJK5
K. pneumoniae 342 B5XVF9
K. oxytoca ATCC 8724 G8WAR6
K. pneumoniae NJST258_2 W8UR02
K. pneumoniae MGH 78578 A6TCS4
K. pneumoniae PittNDM01 A0A075VLH4
K. pneumoniae PittNDM01 A0A075V6L4
K. pneumoniae NJST258_2 W8UKG8
K. pneumoniae MGH 78578 A6T932
K. variicola At-22 D3RFR8
K. pneumoniae 342 B5XQQ8
S. typhimurium SL1344 E1WD68
S. typhimurium LT2 P43661
S. bongori N268-08 S5N1Y7
E. coli E2348/69 B7ULA8
E. albertii KF1 W0AVU6
Y. enterocolitica LC20 W8U8F8
S. bongori N268-08 S5N3Q7
E. coli E24377A A7ZTT8
Eb. lignolyticus SCF1 E3G5Q2
S. bongori N268-08 S5N295
C. koseri ATCC BAA-895 A8APL4
Eb. lignolyticus SCF1 E3G712
Eb. lignolyticus SCF1 E3GBG7
0.2

Figure S2: The evolutionary relationship of the Ush/Yra cluster chaperone sequences. The trees were calculated with (*a*) RAxML, (*b*) MrBayes or (*c*) phylobayes with the C20 model.

Figure S3

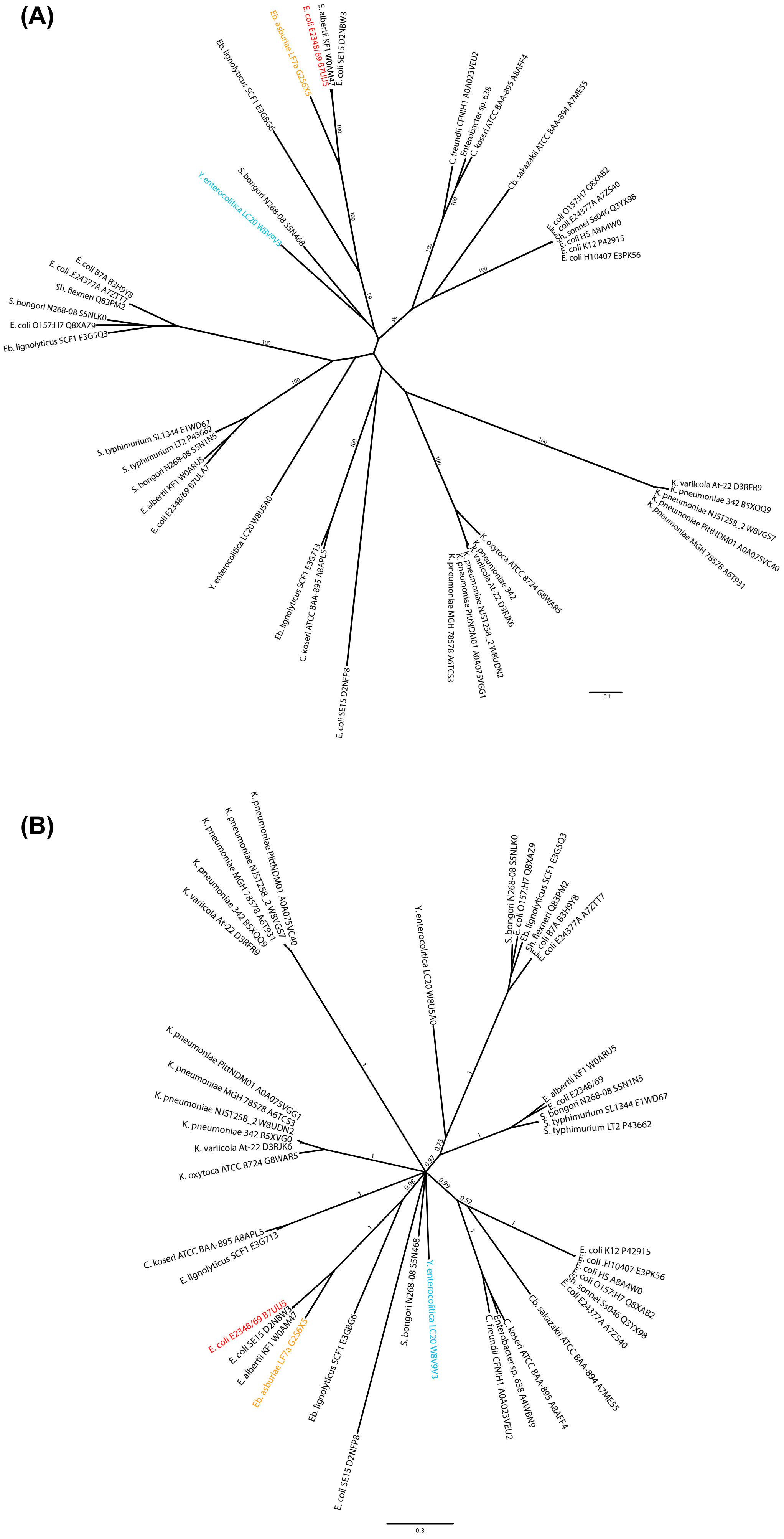
(A)
E. coli SE15 D2NBW3
E. albertii KF1 W0AM47
E. coli E2348/69 B7UIJ5
Eb. asburiae LF7a G2S6X5
Eb. lignolyticus SCF1 E3GBG6
Y. enterocolitica LC20 W8V9V3
S. bongori N268-08 S5N468
C. freundii CFNIH1 A0A023VEU2
Enterobacter sp. 638
C. koseri ATCC BAA-895 A8AFF4
Cb. sakazakii ATCC BAA-894 A7ME55
E. coli O157:H7 Q8XAB2
E. coli E24377A A7ZS40
Sh. sonnei Ss046 Q3YX98
E. coli HS A8A4W0
E. coli K12 P42915
E. coli H10407 E3PK56
E. coli B7A B3H9Y8
E. coli E24377A A7ZTT7
Sh. flexneri Q83PM2
S. bongori N268-08 S5NLK0
E. coli O157:H7 Q8XAZ9
Eb. lignolyticus SCF1 E3G5Q3
S. typhimurium SL1344 E1WD67
S. typhimurium LT2 P43662
S. bongori N268-08 S5N1N5
E. albertii KF1 W0ARU5
E. coli E2348/69 B7ULA7
Y. enterocolitica LC20 W8U5A0
Eb. lignolyticus SCF1 E3G713
C. koseri ATCC BAA-895 A8APL5
E. coli SE15 D2NFP8
K. oxytoca ATCC 8724 G8WAR5
K. pneumoniae 342
K. variicola At-22 D3RJK6
K. pneumoniae NJST258_2 W8UDN2
K. pneumoniae PittNDM01 A0A075VGG1
K. pneumoniae MGH 78578 A6TCS3
K. variicola At-22 D3RFR9
K. pneumoniae 342 B5XQQ9
K. pneumoniae NJST258_2 W8VGS7
K. pneumoniae PittNDM01 A0A075VC40
K. pneumoniae MGH 78578 A6T931
0.1
(B)
K. pneumoniae PittNDM01 A0A075VC40
K. pneumoniae NJST258_2 W8VGS7
K. pneumoniae MGH 78578 A6T931
K. pneumoniae 342 B5XQQ9
K. variicola At-22 D3RFR9
Y. enterocolitica LC20 W8U5A0
S. bongori N268-08 S5NLK0
E. coli O157:H7 Q8XAZ9
Eb. lignolyticus SCF1 E3G5Q3
Sh. flexneri Q83PM2
E. coli B7A B3H9Y8
E. coli E24377A A7ZTT7
K. pneumoniae PittNDM01 A0A075VGG1
K. pneumoniae MGH 78578 A6TCS3
K. pneumoniae NJST258_2 W8UDN2
K. pneumoniae 342 B5XVG0
K. variicola At-22 D3RJK6
K. oxytoca ATCC 8724 G8WAR5
E. albertii KF1 W0ARU5
E. coli E2348/69
S. bongori N268-08 S5N1N5
S. typhimurium SL1344 E1WD67
S. typhimurium LT2 P43662
C. koseri ATCC BAA-895 A8APL5
E. lignolyticus SCF1 E3G713
E. coli K12 P42915
E. coli H10407 E3PK56
E. coli HS A8A4W0
E. coli O157:H7 Q8XAB2
Sh. sonnei Ss046 Q3YX98
E. coli E24377A A7ZS40
E. coli E2348/69 B7UIJ5
E. coli SE15 D2NBW3
E. albertii KF1 W0AM47
Eb. asburiae LF7a G2S6X5
Eb. lignolyticus SCF1 E3GBG6
S. bongori N268-08 S5N468
Y. enterocolitica LC20 W8V9V3
C. freundii CFNIH1 A0A023VEU2
Enterobacter sp. 638 A4WBN9
C. koseri ATCC BAA-895 A8AFF4
Cb. sakazakii ATCC BAA-894 A7ME55
E. coli SE15 D2NFP8
0.3

Figure S3: Unrooted tree representation of figure 2(c). The trees were calculated with RaxML (*a*) or phylobayes with the C60 model (*b*).

Figure S4

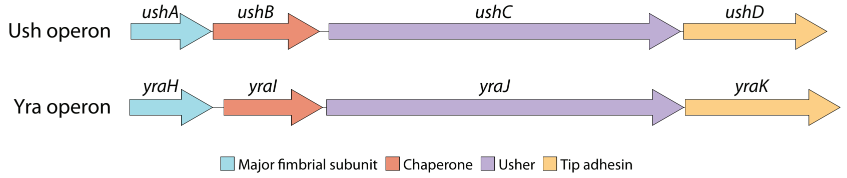
Ush operon
ushA
ushB
ushC
ushD
Yra operon
yraH
yraI
yraJ
yraK
Major fimbrial subunit
Chaperone
Usher
Tip adhesin

Figure S4: Genetic organisation of selected fimbrial operons. Illustrations of the *ush* and *yra* operons from *E. coli* strains SE15 and K-12 substr. MG1655, respectively.

Figure S5

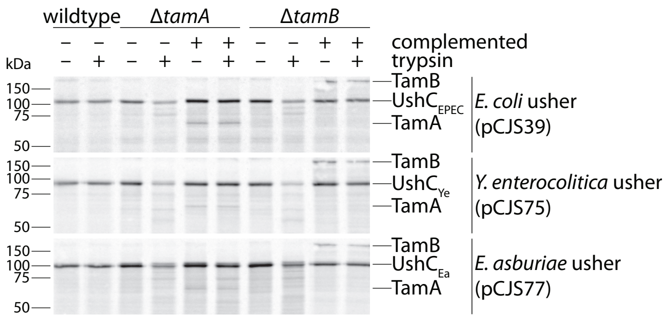
wildtype
ΔtamA
ΔtamB
complemented
trypsin
kDa
150
100
75
50
TamB
UshC EPEC
TamA
E. coli usher
(pCJS39)
UshC Ye
Y. enterocolitica usher
(pCJS75)
UshC Ea
E. asburiae usher
(pCJS77)

Figure S5: Complementation analysis of usher biogenesis in *E. coli*. UshC biogenesis was assessed by pulse chase analysis in *E. coli* cells harbouring the indicated plasmids in conjunction with either the pACYCDuet-1 base vector or a complementation plasmid (indicated by "-" or "+" complemented, respectively): pCJS69 (pACYCDuet-1 containing *tamA*) or pCJS72 (pACYC-Duet-1 containing *tamB*). Aliquots were taken at 16 minutes and treated with ("+") or without ("-") 20µg/mL trypsin (static, 10 min, on ice). Proteins were then TCA-precipitated, washed with acetone and boiled for 10 minutes in SDS sample buffer. Analysis was performed by SDS-PAGE and storage phosphor-imaging.

References supplement

Kelley LA, Sternberg MJ. 2009. Protein structure prediction on the Web: a case study using the Phyre server. Nat Protoc 4:363-371.
Wurpel DJ, Beatson SA, Totsika M, Petty NK, Schembri MA. 2013. Chaperone-usher fimbriae of Escherichia coli. PLoS One 8:e52835.

Table S1: The reference strains used in figure 1.

organism	taxID	proteome ID	Genome assembly	download da	pathovar
Escherichia fergusonii (strain ATCC 35469 / DSM 13698 / CDC 0568-73)	585054	UP000000745	GCA_000026225.1	04.05.2015	commensal
Shimwellia blattae (strain ATCC 29907 / DSM 4481 / JCM 1650 / NBRC 105725 / CDC 9005-74) (*Escherichia blattae*)	630626	UP000001955	GCA_000262305.1	04.05.2015	none
Escherichia coli (strain K12) K12 / MG1655 / ATCC 47076	83333	UP000000625	GCA_000005845.2	04.05.2015	lab-strain
Escherichia coli O9:H4 (strain HS)	331112	UP000001123	GCA_000017765.1	04.05.2015	commensal
Escherichia coli B7A	340184	UP000027984	GCA_000725265.1	04.05.2015	ETEC
Escherichia coli O78:H11 (strain H10407 / ETEC)	316401	UP000006877	GCA_000210475.1	04.05.2015	ETEC
Escherichia coli O139:H28 (strain E24377A / ETEC)	331111	UP000001122	GCA_000017745.1	04.05.2015	ETEC
Escherichia coli O157:H7 (strain Sakai / EHEC)	83334	UP000000558	GCA_000008865.1	04.05.2015	EHEC
Escherichia coli 53638	344610	UP000004441	GCA_000167915.2	04.05.2015	EIEC
Escherichia coli O6:K15:H31 (strain 536 / UPEC)	362663	UP000009182	GCA_000013305.1	04.05.2015	UPEC
Escherichia coli O6:H1 (strain CFT073 / ATCC 700928 / UPEC)	199310	UP000001410	GCA_000007445.1	04.05.2015	UPEC
Escherichia coli (strain UTI89 / UPEC)	364106	UP000001952	GCA_000013265.1	04.05.2015	UPEC
Escherichia albertii KF1	1440052	UP000018871	GCA_000512125.1	07.05.2015	enteric
Escherichia coli O127:H6 (strain E2348/69 / EPEC)	574521	UP000008205	GCA_000026545.1	07.05.2015	EPEC
Escherichia coli O150:H5 (strain SE15)	431946	UP000009157	GCA_000010485.1	07.05.2015	commensal
Shigella flexneri ATCC 700930 / 2457T / Serotype 2a	623	UP000002673	GCA_000007405.1	04.05.2015	enteric
Shigella flexneri 301 / Serotype 2a	623	UP000001006	GCA_000006925.2	04.05.2015	enteric
Shigella sonnei (strain Ss046)	300269	UP000002529	GCA_000092525.1	04.05.2015	enteric
Klebsiella pneumoniae subsp. pneumoniae (strain ATCC 700721 / MGH 78578)	272620	UP000000265	GCA_000016305.1	04.05.2015	
Klebsiella variicola (strain At-22)	640131	UP000001907	GCA_000025465.1	04.05.2015	
Klebsiella pneumoniae (strain 342)	507522	UP000001734	GCA_000019565.1	04.05.2015	
Klebsiella oxytoca (strain ATCC 8724 / DSM 4798 / JCM 20051 / NBRC 3318 / NRRL B-199 / KCTC 1686)	1006551	UP000007843	GCA_000240325.1	04.05.2015	
Klebsiella pneumoniae subsp. pneumoniae PittNDM01	1406314	UP000028499	GCA_000733255.1	04.05.2015	
Klebsiella pneumoniae 30684/NJST258_2	1420013	UP000019586	GCA_000597905.1	04.05.2015	
Citrobacter rodentium (strain ICC168) (*Citrobacter freundii* biotype 4280)	637910	UP000001889	GCA_000027085.1	04.05.2015	
Citrobacter koseri (strain ATCC BAA-895 / CDC 4225-83 / SGSC4696)	290338	UP000008148	GCA_000018045.1	04.05.2015	
Citrobacter freundii CFNIH1	1333848	UP000025226	GCA_000648515.1	04.05.2015	
Enterobacter sp. (strain 638)	399742	UP000000230	GCA_000016325.1	04.05.2015	
Enterobacter lignolyticus (strain SCF1)	701347	UP000006872	GCA_000164865.1	04.05.2015	
Cronobacter sakazakii (strain ATCC BAA-894) (*Enterobacter sakazakii*)	290339	UP000000260	GCA_000017665.1	04.05.2015	
Pluralibacter gergoviae (*Enterobacter gergoviae*)	61647	UP000029498	GCA_000757785.1	04.05.2015	
Enterobacter aerogenes (strain ATCC 13048 / DSM 30053 / JCM 1235 / KCTC 2190 / NBRC 13534 / NCIMB 10102 / NCTC 10006)	1028307	UP000008881	GCA_000215745.1	04.05.2015	
Enterobacter cloacae subsp. cloacae (strain ATCC 13047 / DSM 30054 / NBRC 13535 / NCDC 279-56)	716541	UP000002363	GCA_000025565.1	04.05.2015	
Enterobacter asburiae (strain LF7a)	640513	UP000008527	GCA_000224675.1	07.05.2015	
Yersinia enterocolitica LC20	1443113	UP000019587	GCA_000597945.1	04.05.2015	
Yersinia pestis CO-92 / Biovar Orientalis	632	UP000000815	GCA_000009065.1	04.05.2015	
Yersinia pseudotuberculosis serotype I (strain IP32953)	273123	UP000001011	GCA_000047365.1	04.05.2015	
Yersinia pestis bv. Antiqua (strain Nepal516)	377628	UP000008936	GCA_000013805.1	04.05.2015	
Yersinia pestis biovar Orientalis str. IP275	373665	UP000004430	GCA_000168235.2	04.05.2015	
Yersinia pseudotuberculosis serotype IB (strain PB1/+)	502801	UP000001193	GCA_000020085.1	04.05.2015	
Salmonella typhimurium (strain LT2 / SGSC1412 / ATCC 700720)	99287	UP000001014	GCA_000006945.1	04.05.2015	
Salmonella gallinarum (strain 287/91 / NCTC 13346)	550538	UP000008321	GCA_000009525.1	04.05.2015	
Salmonella bongori N268-08	1197719	UP000015042	GCA_000439255.1	04.05.2015	
Salmonella typhi CT18	90370	UP000000541	GCA_000195995.1	04.05.2015	
Salmonella typhi Ty2	90370	UP000002670	GCA_000007545.1	04.05.2015	
Salmonella paratyphi A (strain AKU_12601)	554290	UP000001869	GCA_000026565.1	04.05.2015	
Salmonella typhimurium (strain SL1344)	216597	UP000008962	GCA_000210855.2	04.05.2015	

Table S2: The accession numbers of the operon sequences as investigated in figure 4, S2 and S3.

organism	accession majo	locus tag ma	accession chape	locus tag cha	accession us	locus tag ush	accession ti	locus tag tip	accession ot	locus tag oth	operon ok?	comment	gene upstream		same strand	gene downstream		same strand?		
Citrobacter freundii CFNIH1	A0A023VD70	CFNIH1_205	A0A023VEK1	CFNIH1_205	A0A023VEU2	CFNIH1_205	A0A023VDU	CFNIH1_20580					CFNIH1_205	Type III expo	no	CFNIH1_205	Chemotaxis	no		
Citrobacter koseri (strain ATCC BAA-895 / CDC 4225-83 / S	A8AFF6	CKO_01076	A8AFF5	CKO_01075	A8AFF4	CKO_01074	A8AFF2	CKO_01073					CKO_01077	unknown		CKO_01071	Chemotaxis	no		
Citrobacter koseri (strain ATCC BAA-895 / CDC 4225-83 / S	A8APL3	CKO_04370	A8APL4	CKO_04371	A8APL5	CKO_04372	A8APL6	CKO_04373					CKO_04369	transcr.reg.		CKO_04374	DNA-binding prot.			
Cronobacter sakazakii (strain ATCC BAA-894) (Enterobacte	A7ME57	ESA_02345	A7ME56	ESA_02344	A7ME55	ESA_02343	A7MFZ9	ESA_02342					ESA_02347	oxidoreductase		ESA_02341	Glyoxylate/hydroxypyruvate reductase			
Enterobacter asburiae (strain LF7a)	G2S6X3	Entas_0744	G2S6X4	Entas_0745	G2S6X5	Entas_0746	G2S6X6	Entas_0747					Entas_0743	Penicillin-binding protein 1		Entas_0748	TonB-dependent receptor			
Enterobacter lignolyticus (strain SCF1)	E3G5Q1	Entcl_4425	E3G5Q2	Entcl_4426	E3G5Q3	Entcl_4427	E3G5Q4	Entcl_4428					Entcl_4424	aminotransferase		Entcl_4429	ABC transporter			
Enterobacter lignolyticus (strain SCF1)	E3G711	Entcl_1063	E3G712	Entcl_1064	E3G713	Entcl_1065	E3G714	Entcl_1066					Entcl_1062	transcr.reg.		Entcl_1067	EAL domain protein			
Enterobacter lignolyticus (strain SCF1)	E3GBG8	Entcl_2683	E3GBG7	Entcl_2682	E3GBG6	Entcl_2681	E3GBG5	Entcl_2680				The fimbrial tip annotated sequen	Entcl_2684	Glycine zipper		Entcl_2679	LysM domain	no		
Enterobacter sp. (strain 638)	A4WBN7	Ent638_2448	A4WBN8	Ent638_2449	A4WBN9	Ent638_2450	A4WBP0	Ent638_2451					Ent638_2447	Type III expo	no	Ent638_2452	Chemotaxis	no		
Escherichia albertii KF1	W0AP51	EAKF1_ch12	W0ASK3	EAKF1_ch12	W0AM47	EAKF1_ch12	W0AS87	EAKF1_ch1264c					EAKF1_ch12	Penicillin-binding protein 1		EAKF1_ch12	TonB-dependent receptor			
Escherichia albertii KF1	W0AQA2	EAKF1_ch23	W0AVU6	EAKF1_ch23	W0ARU5	EAKF1_ch23	W0AV98	EAKF1_ch23	W0AVV0	EAKF1_ch2401		*IS*629 between tip adhesin and thi	EAKF1_ch23	MFS		EAKF1_ch23	transposase			
Escherichia coli (strain K-12 substr. MG1655)	P42913	b3142	P42914	b3143	P42915	b3144	P43319	b3145					b3141	Glucosamine isomerase		b3146	rRNA methyl	no		
Escherichia coli B7A	B3H9Y6	L960_3125	A0A075L8R3-A0	L960_3126-L	B3H9Y8	L960_3128	B3H9Y9	L960_3129				chaperone broken	L960_3124	Glucosamine isomerase		L960_3130	ABC transporter			
Escherichia coli O127:H6 (strain E2348/69 / EPEC)	B7UIJ4	E2348C_015	pseudo		B7UIJ5	E2348C_015	B7UIJ6	E2348C_0156				chaperone broken	E2348C_015	Penicillin-binding protein 1		E2348C_015	TonB-dependent receptor			
Escherichia coli O127:H6 (strain E2348/69 / EPEC)	B7ULA9	E2348C_379	B7ULA8	E2348C_379	B7ULA7	E2348C_379	B7ULA6	E2348C_379	B7ULA5	E2348C_3794			E2348C_379	MFS		E2348C_379	alkaline phosphatase			
Escherichia coli O139:H28 (strain E24377A / ETEC)	A7ZS38	EcE24377A_	A7ZS39	EcE24377A_	A7ZS40	EcE24377A_	A7ZS41	EcE24377A_3627					EcE24377A_	Glucosamine isomerase		EcE24377A_	rRNA methyl	no		
Escherichia coli O139:H28 (strain E24377A / ETEC)	A7ZTT9	EcE24377A_	A7ZTT8	EcE24377A_	A7ZTT7	EcE24377A_	A7ZTT6	EcE24377A_4240					EcE24377A_	Glucosamine isomerase		EcE24377A_	ABC transporter			
Escherichia coli O150:H5 (strain SE15)	D2NBW1	ECSF_0163	D2NBW2	ECSF_0164	D2NBW3	ECSF_0165	D2NBW4	ECSF_0166					ECSF_0162	Penicillin-binding protein 1		ECSF_0167	TonB-dependent receptor			
Escherichia coli O150:H5 (strain SE15)		c(4372031-4	D2NFP9	ECSF_4009	D2NFP8	ECSF_4008						no tip adhesin, manually annotate	ECSF_4010	Peptidase S6	no	ECSF_4007	acyl-CoA acy	no		
Escherichia coli O157:H7 (strain Sakai)	Q8XAB4	Z4498	Q8XAB3	Z4499	Q8XAB2	Z4500	Q8XAB1	Z4501				Tip adhesin truncated	Z4497	Glucosamine isomerase		Z4502-Z4505	transposase-	yes-yes-yes-yes-no		
Escherichia coli O157:H7 (strain Sakai)	Q8XAZ0	Z5225	Q8XAZ6-Q8XAZ7	Z5224-Z5223	Q8XAZ9	Z5222	Q8XB01	Z5221	Q8XB02	Z5220		chaperone truncated	Z5226-Z5227	tiny-Glucosamine isomera		Z5219	ABC transporter			
Escherichia coli O78:H11 (strain H10407 / ETEC)	E3PK54	ETEC_3409	E3PK55	ETEC_3410	E3PK56	ETEC_3411	E3PK57	ETEC_3412					ETEC_3408	Glucosamine isomerase		ETEC_3413	rRNA methyl	no		
Escherichia coli O9:H4 (strain HS)	A8A4V8	EcHS_A3334	A8A4V9	EcHS_A3335	A8A4W0	EcHS_A3336	A8A4W1	EcHS_A3337					EcHS_A3333	Glucosamine isomerase		EcHS_A3338	rRNA methyl	no		
Klebsiella oxytoca (*K. michiganensis*) (strain ATCC 8724 /	G8WAR7	KOX_00365	G8WAR6	KOX_00360	G8WAR5	KOX_00355	G8WAR4	KOX_00350					KOX_00375-	tiny-aldehyde dehydrogen		KOX_00345-	hexapeptide transferase - solute-binding protein			
Klebsiella pneumoniae (strain 342)	B5XQQ7	KPK_2701	B5XQQ8	KPK_2702	B5XQQ9	KPK_2703	B5XQR0	KPK_2704					KPK_2700	unknown	no	KPK_2705	glycoside hydrolase			
Klebsiella pneumoniae (strain 342)	B5XVF7	KPK_1142	B5XVF9	KPK_1144	B5XVG0	KPK_1145	B5XVG1	KPK_1147				A small ORF was annotated betwe	KPK_1141-KI	tiny-aldehyde dehydrogen		KPK_1146-KI	chap-adh-sol	no-no-yes		
Klebsiella pneumoniae 30684/NJST258_2	W8UVV7	KPNJ2_0128	W8UR02	KPNJ2_0128	W8UDN2	KPNJ2_0128	W8UG85	KPNJ2_01285					KPNJ2_0128	aldehyde dehydrogenase		KPNJ2_0128	adh-usher-ch	no-no-no-no-no-no-yes		a tiny protein in between above
Klebsiella pneumoniae 30684/NJST258_2	W8UI31	KPNJ2_0278	W8UKG8	KPNJ2_0279	W8VGS7	KPNJ2_0279	W8V048	KPNJ2_02792					KPNJ2_0278	unknown	no	KPNJ2_0279	glycoside hydrolase			
Klebsiella pneumoniae subsp. pneumoniae (strain ATCC 70	A6T933	KPN_01673	A6T932	KPN_01672	A6T931	KPN_01671	A6T930	KPN_01670					KPN_01674	Peptidase M24		KPN_01669	glycoside hydrolase		looks like unknown protein as in seuqences above is just not called	
Klebsiella pneumoniae subsp. pneumoniae (strain ATCC 70	A6TCS5	KPN_02990	A6TCS4	KPN_02989	A6TCS3	KPN_02988	A6TCS2	KPN_02987				second fimbrial operon next to it;	KPN_02991	aldehyde dehydrogenase		KPN_02986-	usher-chap-a	no-no-no-no-yes		
Klebsiella pneumoniae subsp. pneumoniae PittNDM01	A0A075V8V7	Q770_05925	A0A075V6L4	Q770_05920	A0A075VC40	Q770_05915	A0A075VG0	Q770_05910					Q770_05930	unknown	no	Q770_05905	glycoside hydrolase			
Klebsiella pneumoniae subsp. pneumoniae PittNDM01	A0A075VR62	Q770_25190	A0A075VLH4	Q770_25195	A0A075VGG	Q770_25200	A0A075VJ9	Q770_25205				second fimbrial operon next to it;	Q770_25185	aldehyde dehydrogenase		Q770_25210	adh-usher-ch	no-no-no-no-no-yes		
Klebsiella variicola (strain At-22)	D3RFR7	Kvar_2655	D3RFR8	Kvar_2656	D3RFR9	Kvar_2657	D3RFS0	Kvar_2658					Kvar_2654	unknown	no	Kvar_2659	glycoside hydrolase			
Klebsiella variicola (strain At-22)	D3RJK4	Kvar_1080	D3RJK5	Kvar_1081	D3RJK6	Kvar_1082	D3RJK7	Kvar_1083					Kvar_1079	aldehyde dehydrogenase		Kvar_1084-K	chap-adh-sol	no-no-yes		
Salmonella bongori N268-08	S5NE28	A464_3721	S5N1Y7	A464_3720	S5N1N5	A464_3719	S5NKU9	A464_3718	S5MW79	A464_3717			A464_3722	MFS?		A464_3716	alkaline phosphatase			
Salmonella bongori N268-08	S5NN90	A464_4500	S5N3Q7	A464_4501	S5N468	A464_4502	S5NGE2	A464_4503					A464_4499	integrase		A464_4504	unknown			
Salmonella bongori N268-08	S5N2J6	A464_3925	S5N295	A464_3924	S5NLK0	A464_3923	S5MWU1	A464_3922					A464_3926-A	tiny-Glucosamine isomera		A464_3921	ABC transporter			
Salmonella typhimurium (strain LT2 / SGSC1412 / ATCC 70	P43660	STM3640	P43661	STM3639	P43662	STM3638	P43663	STM3637	P43664	STM3636			STM3641	autotransporter YhjY		STM3635	alkaline phosphatase			
Salmonella typhimurium (strain SL1344)	E1WD69	SL1344_3606	E1WD68	SL1344_3605	E1WD67	SL1344_3604	pseudogene	SL1344_3602	E1WD65	SL1344_3601		adhesin II is a pseudogene	SL1344_3607	autotransporter YhjY		SL1344_3600	alkaline phosphatase			
Shigella flexneri 2a str. 2457T					Q83PM2	SF3723	Q83J02	SF3726				IS elements between usher & tip a	SF3722	transposase	no	SF3727	ABC transporter			
Shigella sonnei (strain Ss046)					Q3YX98	SSON_3289	Q3YX96	SSON_3291				IS elements between usher & tip a	SSON_3288	transposase	no	SSON_3292	rRNA methyl	no	SSON_3290	integrase between usher and fimII
Yersinia enterocolitica LC20	W8UMD5	LC20_03798	W8V9B9-W8U7	LC20_03797-	W8U5A0	LC20_03795	W8UHH1	LC20_03794	W8UMC9	LC20_03793		chaperone broken	LC20_03799	phna family	no	LC20_03792-	tiny-ATPase	no-yes		
Yersinia enterocolitica LC20	W8U601	LC20_04040	W8U8F8	LC20_04041	W8V9V3	LC20_04042	W8UN19	LC20_04043				Tip adhesin truncated; fimbrial ush	LC20_04039-	unknown-un	no-no-yes	LC20_04044	unknown		two tiny proteins upstream, therefore 3 annotated	

Table S3: The accession numbers of the strains used to investigate the E. coli usher diversity as shown in figure 1 and 2.

organism	strain	assembly	pathovar	ST	classification comments	
Escherichia coli	MG1655	GCA_000005845.2	LS	10		
Escherichia coli	EDL933	GCA_000006665.1	EHEC	66	STX1, ST2	
Escherichia coli	CFT073	GCA_000007445.1	UPEC	73		
Escherichia coli	Sakai	GCA_000008865.1	EHEC	66	STX1, STX2	
Escherichia coli	K12-W3110	GCA_000010245.1	LS	10		
Escherichia coli	SE11	GCA_000010385.1	C	156		
Escherichia coli	12009	GCA_000010745.1	EHEC	119	STX1, STX2, NO BFP	LS = laboratory strain
Escherichia coli	11128	GCA_000010765.1	EHEC	106	STX1, STX2	EHEC = enterohaemorrhagic *E. coli* - LEE-positive, STX-positive, BFP-negative
Escherichia coli	UTI89	GCA_000013265.1	UPEC	NA		UPEC = uropathogenic *E. coli*
Escherichia coli	536	GCA_000013305.1	UPEC	4727		C = commensal
Escherichia coli	E24377A	GCA_000017745.1	UPEC	1132		other = see details in classification comments
Escherichia coli	HS	GCA_000017765.1	C	46		ATEC = atypical EPEC - LEE-positive, STX-negative, BFP-negative (or pseudogenised bfpA)
Escherichia coli	B_REL606	GCA_000017985.1	LS	93		EPEC = enteropathogenic E. coli - LEE-positive, STX-negative, BFP-positive
Escherichia coli	ATCC 8739	GCA_000019385.1	LS	NA		EAEC = enteroaggregative E. coli
Escherichia coli	DH10B	GCA_000019425.1	LS	1060		ExPEC = extraintestinal pathogenic E. coli
Escherichia coli	SMS-3-5	GCA_000019645.1	other	354	Environmental isolate	
Escherichia coli	EC4115	GCA_000021125.1	EHEC	66	STX2	
Escherichia coli	TW14359	GCA_000022225.1	EHEC	66	STX2	
Escherichia coli	BW2952	GCA_000022345.1	LS	10		
Escherichia coli	BL21-DE3	GCA_000022665.2	LS	93		
Escherichia coli	DH1	GCA_000023365.1	LS	1060		
Escherichia coli	BL21	GCA_000023665.1	LS	93		
Escherichia coli	CB9615	GCA_000025165.1	ATEC	73	no BFP no STX	
Escherichia coli	IHE3034	GCA_000025745.1	ExPEC	95		
Escherichia coli	55989	GCA_000026245.1	EAEC	678		
Escherichia coli	IAI1	GCA_000026265.1	C	1128		
Escherichia coli	S88	GCA_000026285.1	ExPEC	95		
Escherichia coli	ED1a	GCA_000026305.1	C	4728		
Escherichia coli	UMN026	GCA_000026325.1	UPEC	597		
Escherichia coli	IAI39	GCA_000026345.1	ExPEC	62		
Escherichia coli	E2348/69	GCA_000026545.1	EPEC	18	BFP	
Escherichia coli	042	GCA_000027125.1	EAEC	NA		
Escherichia coli	11368	GCA_000091005.1	EHEC	106	STX1	
Escherichia coli	KO11FL	GCA_000147855.3	LS	1079		
Escherichia coli	ABU83972	GCA_000148365.1	other	73	Asymptomatic bactiuria isolate	
Escherichia coli	UM146	GCA_000148605.1	other	643	Adherent invasive E. coli	
Escherichia coli	B7A	GCA_000167815.1	ETEC	94		
Escherichia coli	2362-75	GCA_000183005.2	EPEC	18	BFP	
Escherichia coli	NRG857C	GCA_000183345.1	other	135	Adherent invasive E. coli	
Escherichia coli	W	GCA_000184185.1	LS	1079		
Escherichia coli	EHEC14	GCA_000188755.2	EHEC	106	stx1	
Escherichia coli	E128010	GCA_000188775.2	EPEC	118	BFP	
Escherichia coli	OK1180	GCA_000188835.2	EHEC	106	STX1, STX2	
Escherichia coli	RN587/1	GCA_000188875.2	EPEC	14		
Escherichia coli	H10407	GCA_000210475.1	ETEC	48		
Escherichia coli	UMNK88	GCA_000212715.2	ETEC	100		
Escherichia coli	NA114	GCA_000214765.2	UPEC	131		
Escherichia coli	TY-2482	GCA_000217695.2	EAEC	678		
Escherichia coli	UMNF18	GCA_000220005.1	ETEC	10		
Escherichia coli	H.I.8.	GCA_000225185.2	other	119	VTEC; STX+; BFP+	
Escherichia coli	CE10	GCA_000227625.1	ExPEC	62	NMEC	
Escherichia coli	CloneDi2	GCA_000233875.1	UPEC	73		
Escherichia coli	cloneDi14	GCA_000233895.1	UPEC	73		
Escherichia coli	RM12579	GCA_000245515.1	ATEC	335	NO BFP (manual inspection of 5 plasmids)	
Escherichia coli	DEC1A	GCA_000249055.2	EPEC	18	BFP	
Escherichia coli	DEC1B	GCA_000249075.2	EPEC	18	BFP	
Escherichia coli	DEC1C	GCA_000249095.2	EPEC	18	BFP	
Escherichia coli	DEC1D	GCA_000249115.2	EPEC	18	BFP	
Escherichia coli	DEC1E	GCA_000249135.2	EPEC	18	BFP	
Escherichia coli	DEC2A	GCA_000249155.2	EPEC	18	BFP	
Escherichia coli	DEC2B	GCA_000249175.2	EPEC	18	BFP	
Escherichia coli	DEC2C	GCA_000249195.2	EPEC	18	BFP	
Escherichia coli	DEC2D	GCA_000249215.2	EPEC	18	BFP	
Escherichia coli	DEC2E	GCA_000249235.2	EPEC	18	BFP	
Escherichia coli	DEC4A	GCA_000249375.2	ATEC	66	no BFP no STX	
Escherichia coli	DEC4B	GCA_000249395.2	EHEC	66	stx2	
Escherichia coli	DEC4C	GCA_000249415.2	ATEC	66	no BFP no STX	
Escherichia coli	DEC4D	GCA_000249435.2	EHEC	66	STX2	
Escherichia coli	DEC4E	GCA_000249455.2	EHEC	66	STX1	
Escherichia coli	DEC5A	GCA_000249495.2	ATEC	73	NO BFP no STX	
Escherichia coli	DEC5B	GCA_000249515.2	ATEC	73	NO BFP no STX	
Escherichia coli	DEC5C	GCA_000249535.2	ATEC	73	NO BFP no STX	
Escherichia coli	DEC5D	GCA_000249555.2	ATEC	73	NO BFP no STX	
Escherichia coli	DEC5E	GCA_000249575.2	ATEC	73	NO BFP no STX	
Escherichia coli	DEC9A	GCA_000249895.2	ATEC	106	NO BFP no STX	
Escherichia coli	DEC9B	GCA_000249915.2	ATEC	106	NO BFP no STX	
Escherichia coli	DEC9C	GCA_000249935.2	ATEC	106	NO BFP no STX	
Escherichia coli	DEC9D	GCA_000249955.2	ATEC	106	NO BFP no STX	
Escherichia coli	DEC9E	GCA_000249975.2	ATEC	106	NO BFP no STX	
Escherichia coli	DEC11A	GCA_000250115.2	ATEC	119	NO BFP no STX	
Escherichia coli	DEC11B	GCA_000250135.2	ATEC	119	NO BFP no STX	
Escherichia coli	DEC11C	GCA_000250155.2	EHEC	119	STX1 NO BFP	
Escherichia coli	DEC11D	GCA_000250175.2	ATEC	118	pseudogenised bfpA	
Escherichia coli	DEC11E	GCA_000250195.2	ATEC	118	pseudogenised bfpA	
Escherichia coli	DEC12A	GCA_000250215.2	ATEC	118	NO BFP no STX	
Escherichia coli	DEC12B	GCA_000250235.2	EPEC	118	BFP	
Escherichia coli	DEC12C	GCA_000250255.2	EPEC	NA	BFP	
Escherichia coli	DEC12D	GCA_000250275.2	EPEC	118	BFP	
Escherichia coli	DEC12E	GCA_000250295.2	EPEC	118	BFP	
Escherichia coli	p12b	GCA_000257275.1	other	10	unclear designation	
Escherichia coli	Xuzhou21	GCA_000262125.1	EHEC	11		
Escherichia coli	LF82	GCA_000284495.1	other	135	AIEC	
Escherichia coli	2009EL-2050	GCA_000299255.1	EAEC	0		
Escherichia coli	2009EL-2071	GCA_000299475.1	EAEC	678		
Escherichia coli	APEC_O78	GCA_000332755.1	other	23	APEC	
Escherichia coli	K12-MDS42	GCA_000350185.1	LS	10		
Escherichia coli	C639-08	GCA_000410655.2	ATEC	14	NO BFP no STX	
Escherichia coli	C844-97	GCA_000410675.2	ATEC	14	NO BFP no STX	
Escherichia coli	C12-92	GCA_000446075.2	ATEC	73	NO BFP no STX	
Escherichia coli	C1244-91	GCA_000446095.2	ATEC	567	NO BFP no STX	
Escherichia coli	C154-11	GCA_000446135.2	ATEC	73	NO BFP no STX	
Escherichia coli	C157-11	GCA_000446175.2	ATEC	73	NO BFP no STX	
Escherichia coli	C161-11	GCA_000446195.2	ATEC	78	NO BFP no STX	
Escherichia coli	C213-10	GCA_000446225.2	ATEC	171	NO BFP no STX	
Escherichia coli	OK1114	GCA_000446245.1	EHEC	106	STX1, STX2	
Escherichia coli	C166-11	GCA_000446265.2	ATEC	106	NO BFP no STX	
Escherichia coli	C2139-99	GCA_000446285.2	ATEC	NA	NO BFP no STX	
Escherichia coli	C260-92	GCA_000446325.2	ATEC	171	NO BFP no STX	
Escherichia coli	C262-10	GCA_000446345.2	ATEC	541	NO BFP no STX	
Escherichia coli	C283-09	GCA_000446365.2	ATEC	NA	NO BFP no STX	
Escherichia coli	C295-10	GCA_000446385.2	ATEC	13	NO BFP no STX	
Escherichia coli	C341-10	GCA_000446405.2	ATEC	119	NO BFP no STX	
Escherichia coli	C343-08	GCA_000446425.2	ATEC	NA	NO BFP no STX	
Escherichia coli	C347-93	GCA_000446445.2	ATEC	NA	NO BFP no STX	
Escherichia coli	C354-03B	GCA_000446485.2	ATEC	66	NO BFP no STX	
Escherichia coli	C40-11	GCA_000446505.2	ATEC	176	NO BFP no STX	
Escherichia coli	C418-89	GCA_000446525.2	ATEC	283	NO BFP no STX	
Escherichia coli	C458-10	GCA_000446545.2	ATEC	116	NO BFP no STX	
Escherichia coli	C496-10	GCA_000446585.2	ATEC	171	NO BFP no STX	
Escherichia coli	C497-10	GCA_000446605.2	ATEC	NA	pseudogenised bfpA	
Escherichia coli	C527-94	GCA_000446625.2	ATEC	140	NO BFP no STX	
Escherichia coli	C581-05	GCA_000446645.2	EPEC	NA	BFP	
Escherichia coli	C58-11	GCA_000446665.2	ATEC	620	NO BFP no STX	
Escherichia coli	C586-05	GCA_000446685.2	EPEC	NA	BFP	
Escherichia coli	C652-10	GCA_000446705.2	ATEC	254	pseudogenised bfpA	
Escherichia coli	C654-09	GCA_000446725.2	ATEC	106	NO BFP no STX	
Escherichia coli	C717-10	GCA_000446745.2	ATEC	NA	NO BFP no STX	
Escherichia coli	C725-88	GCA_000446765.2	EPEC	205	BFP	
Escherichia coli	C732-98	GCA_000446785.2	ATEC	543	pseudogenised bfpA	
Escherichia coli	C743-03	GCA_000446805.2	ATEC	254	NO BFP no STX	
Escherichia coli	C751-03	GCA_000446825.2	ATEC	433	NO BFP no STX	
Escherichia coli	C78-09C	GCA_000446845.2	ATEC	539	NO BFP no STX	
Escherichia coli	C79-08	GCA_000446865.2	ATEC	NA	NO BFP no STX	
Escherichia coli	C796-10	GCA_000446905.2	ATEC	228	NO BFP no STX	

Escherichia coli	C799-92	GCA_000446925.2	ATEC	106	NO BFP no STX	
Escherichia coli	C80-08	GCA_000446945.2	EPEC	205	BFP	
Escherichia coli	C807-09	GCA_000446965.2	ATEC	311	NO BFP no STX	
Escherichia coli	C82-11	GCA_000446985.2	ATEC	119	NO BFP no STX	
Escherichia coli	C824-10	GCA_000447005.2	ATEC	881	NO BFP no STX	
Escherichia coli	C842-97	GCA_000447025.2	ATEC	66	NO BFP no STX	
Escherichia coli	C87-11	GCA_000447045.2	ATEC	311	pseudogenised bfpA	
Escherichia coli	C887-10	GCA_000447065.2	ATEC	NA	NO BFP no STX	
Escherichia coli	C900-01	GCA_000447085.2	ATEC	119	NO BFP no STX	
Escherichia coli	C93-11	GCA_000447105.2	ATEC	NA	NO BFP no STX	
Escherichia coli	C9-92	GCA_000447125.2	ATEC	NA	NO BFP no STX	
Escherichia coli	C170-11	GCA_000447145.2	ATEC	119	NO BFP no STX	
Escherichia coli	86-24	GCA_000478215.1	EHEC	66	STX2	
Escherichia coli	upec-80	GCA_000776035.1	UPEC	127		
Escherichia coli	upec-23	GCA_000776045.1	UPEC	10		
Escherichia coli	upec-62	GCA_000776055.1	UPEC	421		
Escherichia coli	upec-78	GCA_000776065.1	UPEC	3693		
Escherichia coli	upec-58	GCA_000776115.1	UPEC	83		
Escherichia coli	upec-81	GCA_000776135.1	UPEC	453		
Escherichia coli	upec-77	GCA_000776155.1	UPEC	404		
Escherichia coli	upec-7	GCA_000776175.1	UPEC	95		
Escherichia coli	upec-8	GCA_000776195.1	UPEC	95		
Escherichia coli	upec-84	GCA_000776215.1	UPEC	127		
Escherichia coli	upec-76	GCA_000776235.1	UPEC	95		
Escherichia coli	upec-99	GCA_000776255.1	UPEC	NA		
Escherichia coli	upec-82	GCA_000776285.1	UPEC	69		
Escherichia coli	upec-98	GCA_000776315.1	UPEC	95		
Escherichia coli	upec-69	GCA_000776335.1	UPEC	69		
Escherichia coli	upec-83	GCA_000776355.1	UPEC	NA		
Escherichia coli	upec-73	GCA_000776375.1	UPEC	95		
Escherichia coli	upec-97	GCA_000776395.1	UPEC	131		
Escherichia coli	upec-79	GCA_000776415.1	UPEC	968		
Escherichia coli	upec-95	GCA_000776435.1	UPEC	491		
Escherichia coli	upec-85	GCA_000776455.1	UPEC	73		
Escherichia coli	upec-60	GCA_000776505.1	UPEC	127		
Escherichia coli	upec-88	GCA_000776535.1	UPEC	131		
Escherichia coli	upec-61	GCA_000776565.1	UPEC	95		
Escherichia coli	upec-74	GCA_000776595.1	UPEC	131		
Escherichia coli	upec-65	GCA_000776615.1	UPEC	80		
Escherichia coli	upec-89	GCA_000776635.1	UPEC	1159		
Escherichia coli	upec-75	GCA_000776655.1	UPEC	95		
Escherichia coli	upec-59	GCA_000776675.1	UPEC	569		
Escherichia coli	upec-93	GCA_000776695.1	UPEC	73		
Escherichia coli	upec-229	GCA_000776715.1	UPEC	73		
Escherichia coli	upec-94	GCA_000776735.1	UPEC	95		
Escherichia coli	upec-87	GCA_000776745.1	UPEC	372		
Escherichia coli	upec-56	GCA_000776775.1	UPEC	69		
Escherichia coli	upec-9	GCA_000776795.1	UPEC	73		
Escherichia coli	upec-70	GCA_000776815.1	UPEC	1159		
Escherichia coli	upec-90	GCA_000776835.1	UPEC	131		
Escherichia coli	upec-91	GCA_000776855.1	UPEC	127		
Escherichia coli	upec-55	GCA_000776915.1	UPEC	NA		
Escherichia coli	upec-54	GCA_000776925.1	UPEC	131		
Escherichia coli	upec-51	GCA_000776965.1	UPEC	95		
Escherichia coli	upec-50	GCA_000776995.1	UPEC	131		
Escherichia coli	upec-48	GCA_000777025.1	UPEC	95		
Escherichia coli	upec-45	GCA_000777065.1	UPEC	NA		
Escherichia coli	upec-41	GCA_000777095.1	UPEC	NA		
Escherichia coli	upec-40	GCA_000777135.1	UPEC	12		
Escherichia coli	upec-4	GCA_000777155.1	UPEC	131		
Escherichia coli	upec-39	GCA_000777165.1	UPEC	73		
Escherichia coli	upec-38	GCA_000777195.1	UPEC	555		
Escherichia coli	upec-36	GCA_000777215.1	UPEC	127		
Escherichia coli	upec-33	GCA_000777285.1	UPEC	906		
Escherichia coli	upec-31	GCA_000777325.1	UPEC	1844		
Escherichia coli	upec-30	GCA_000777355.1	UPEC	297		
Escherichia coli	upec-3	GCA_000777375.1	UPEC	162		
Escherichia coli	upec-29	GCA_000777395.1	UPEC	131		
Escherichia coli	upec-289	GCA_000777415.1	UPEC	73		
Escherichia coli	upec-288	GCA_000777435.1	UPEC	73		
Escherichia coli	upec-287	GCA_000777455.1	UPEC	73		
Escherichia coli	upec-286	GCA_000777475.1	UPEC	62		
Escherichia coli	upec-285	GCA_000777495.1	UPEC	12		
Escherichia coli	upec-282	GCA_000777535.1	UPEC	10		
Escherichia coli	upec-281	GCA_000777555.1	UPEC	131		
Escherichia coli	upec-28	GCA_000777585.1	UPEC	NA		
Escherichia coli	upec-277	GCA_000777605.1	UPEC	127		
Escherichia coli	upec-276	GCA_000777625.1	UPEC	95		
Escherichia coli	upec-274	GCA_000777655.1	UPEC	3672		
Escherichia coli	upec-273	GCA_000777675.1	UPEC	167		
Escherichia coli	upec-271	GCA_000777695.1	UPEC	131		
Escherichia coli	upec-269	GCA_000777735.1	UPEC	216		
Escherichia coli	upec-266	GCA_000777815.1	UPEC	1159		
Escherichia coli	upec-261	GCA_000777845.1	UPEC	73		
Escherichia coli	upec-260	GCA_000777895.1	UPEC	73		
Escherichia coli	upec-259	GCA_000777945.1	UPEC	131		
Escherichia coli	upec-258	GCA_000777975.1	UPEC	404		
Escherichia coli	upec-256	GCA_000777995.1	UPEC	69		
Escherichia coli	upec-257	GCA_000778015.1	UPEC	1193		
Escherichia coli	upec-255	GCA_000778035.1	UPEC	95		
Escherichia coli	upec-254	GCA_000778055.1	UPEC	69		
Escherichia coli	upec-253	GCA_000778075.1	UPEC	73		
Escherichia coli	upec-251	GCA_000778095.1	UPEC	73		
Escherichia coli	upec-250	GCA_000778105.1	UPEC	95		
Escherichia coli	upec-249	GCA_000778135.1	UPEC	95		
Escherichia coli	upec-248	GCA_000778175.1	UPEC	131		
Escherichia coli	upec-244	GCA_000778215.1	UPEC	83		
Escherichia coli	upec-243	GCA_000778235.1	UPEC	1946		
Escherichia coli	upec-239	GCA_000778275.1	UPEC	372		
Escherichia coli	upec-24	GCA_000778285.1	UPEC	2967		
Escherichia coli	upec-238	GCA_000778315.1	UPEC	1249		
Escherichia coli	upec-237	GCA_000778335.1	UPEC	73		
Escherichia coli	upec-236	GCA_000778355.1	UPEC	2619		
Escherichia coli	upec-235	GCA_000778375.1	UPEC	421		
Escherichia coli	upec-233	GCA_000778395.1	UPEC	421		
Escherichia coli	upec-232	GCA_000778415.1	UPEC	73		
Escherichia coli	upec-230	GCA_000778435.1	UPEC	73		
Escherichia coli	upec-209	GCA_000778465.1	UPEC	95		
Escherichia coli	upec-212	GCA_000778475.1	UPEC	131		
Escherichia coli	upec-207	GCA_000778485.1	UPEC	12		
Escherichia coli	upec-205	GCA_000778545.1	UPEC	224		
Escherichia coli	upec-208	GCA_000778565.1	UPEC	420		
Escherichia coli	upec-211	GCA_000778585.1	UPEC	405		
Escherichia coli	upec-22	GCA_000778635.1	UPEC	48		
Escherichia coli	upec-226	GCA_000778685.1	UPEC	127		
Escherichia coli	upec-220	GCA_000778705.1	UPEC	131		
Escherichia coli	upec-219	GCA_000778725.1	UPEC	91		
Escherichia coli	blood-10-1362	GCA_000778745.1	ExPEC	1249		
Escherichia coli	blood-10-1310	GCA_000778765.1	ExPEC	80		
Escherichia coli	upec-213	GCA_000778785.1	UPEC	3752		
Escherichia coli	upec-221	GCA_000778895.1	UPEC	38		
Escherichia coli	blood-10-1308	GCA_000778915.1	ExPEC	80		
Escherichia coli	upec-227	GCA_000778935.1	UPEC	1193		
Escherichia coli	upec-225	GCA_000778955.1	UPEC	127		
Escherichia coli	blood-10-1227	GCA_000778965.1	ExPEC	1249		
Escherichia coli	blood-10-1009	GCA_000779005.1	ExPEC	10		
Escherichia coli	blood-10-1105	GCA_000779025.1	ExPEC	73		
Escherichia coli	upec-101	GCA_000779055.1	UPEC	420		
Escherichia coli	blood-2011-0141	GCA_000779075.1	ExPEC	393		
Escherichia coli	blood-90544	GCA_000779095.1	ExPEC	405		
Escherichia coli	blood-10-1126	GCA_000779125.1	ExPEC	73		
Escherichia coli	blood-90187	GCA_000779155.1	ExPEC	10		
Escherichia coli	blood-10-1215	GCA_000779175.1	ExPEC	1249		
Escherichia coli	upec-100	GCA_000779215.1	UPEC	372		
Escherichia coli	upec-201	GCA_000779255.1	UPEC	12		
Escherichia coli	blood-11-0254	GCA_000779265.1	ExPEC	131		

Escherichia coli	blood-9-0292	GCA_000779275.1	ExPEC	405		
Escherichia coli	upec-202	GCA_000779295.1	UPEC	28		
Escherichia coli	upec-200	GCA_000779335.1	UPEC	543		
Escherichia coli	blood-90543	GCA_000779365.1	ExPEC	405		
Escherichia coli	upec-197	GCA_000779425.1	UPEC	95		
Escherichia coli	blood-10-1365	GCA_000779455.1	ExPEC	73		
Escherichia coli	upec-196	GCA_000779475.1	UPEC	297		
Escherichia coli	blood-11-0041	GCA_000779495.1	ExPEC	NA		
Escherichia coli	upec-195	GCA_000779515.1	UPEC	537		
Escherichia coli	upec-193	GCA_000779545.1	UPEC	73		
Escherichia coli	upec-186	GCA_000779585.1	UPEC	127		
Escherichia coli	blood-10-1386	GCA_000779615.1	ExPEC	73		
Escherichia coli	upec-188	GCA_000779635.1	UPEC	491		
Escherichia coli	blood-2011-0240	GCA_000779655.1	ExPEC	101		
Escherichia coli	upec-187	GCA_000779675.1	UPEC	69		
Escherichia coli	upec-185	GCA_000779695.1	UPEC	95		
Escherichia coli	upec-184	GCA_000779715.1	UPEC	372		
Escherichia coli	blood-B11-136	GCA_000779745.1	ExPEC	131		
Escherichia coli	blood-10-1607	GCA_000779775.1	ExPEC	131		
Escherichia coli	upec-181	GCA_000779795.1	UPEC	73		
Escherichia coli	upec-180	GCA_000779815.1	UPEC	929		
Escherichia coli	upec-179	GCA_000779835.1	UPEC	144		
Escherichia coli	upec-177	GCA_000779875.1	UPEC	131		
Escherichia coli	upec-176	GCA_000779895.1	UPEC	131		
Escherichia coli	upec-175	GCA_000779915.1	UPEC	130		
Escherichia coli	blood-10-1610	GCA_000779935.1	ExPEC	131		
Escherichia coli	upec-173	GCA_000779955.1	UPEC	10		
Escherichia coli	blood-10-180	GCA_000779975.1	ExPEC	38		
Escherichia coli	upec-172	GCA_000779995.1	UPEC	127		
Escherichia coli	upec-171	GCA_000780015.1	UPEC	131		
Escherichia coli	upec-170	GCA_000780035.1	UPEC	409		
Escherichia coli	blood-2011-0238	GCA_000780075.1	ExPEC	101		
Escherichia coli	upec-169	GCA_000780095.1	UPEC	95		
Escherichia coli	upec-166	GCA_000780115.1	UPEC	12		
Escherichia coli	upec-162	GCA_000780135.1	UPEC	131		
Escherichia coli	upec-161	GCA_000780155.1	UPEC	127		
Escherichia coli	upec-159	GCA_000780175.1	UPEC	131		
Escherichia coli	upec-158	GCA_000780195.1	UPEC	12		
Escherichia coli	upec-157	GCA_000780215.1	UPEC	95		
Escherichia coli	upec-156	GCA_000780235.1	UPEC	127		
Escherichia coli	blood-90145	GCA_000780255.1	ExPEC	131		
Escherichia coli	blood-11-0031	GCA_000780275.1	ExPEC	95		
Escherichia coli	upec-155	GCA_000780295.1	UPEC	69		
Escherichia coli	upec-154	GCA_000780315.1	UPEC	70		
Escherichia coli	upec-153	GCA_000780335.1	UPEC	80		
Escherichia coli	upec-151	GCA_000780355.1	UPEC	58		
Escherichia coli	blood-B-11-184	GCA_000780375.1	ExPEC	131		
Escherichia coli	blood-11-0067	GCA_000780395.1	ExPEC	131		
Escherichia coli	upec-150	GCA_000780415.1	UPEC	491		
Escherichia coli	upec-15	GCA_000780435.1	UPEC	131		
Escherichia coli	blood-11-0068	GCA_000780455.1	ExPEC	131		
Escherichia coli	upec-149	GCA_000780475.1	UPEC	1193		
Escherichia coli	upec-148	GCA_000780495.1	UPEC	131		
Escherichia coli	upec-147	GCA_000780535.1	UPEC	88		
Escherichia coli	upec-146	GCA_000780555.1	UPEC	88		
Escherichia coli	upec-145	GCA_000780575.1	UPEC	69		
Escherichia coli	upec-144	GCA_000780595.1	UPEC	95		
Escherichia coli	upec-143	GCA_000780615.1	UPEC	131		
Escherichia coli	upec-142	GCA_000780635.1	UPEC	131		
Escherichia coli	upec-137	GCA_000780655.1	UPEC	354		
Escherichia coli	upec-14	GCA_000780675.1	UPEC	12		
Escherichia coli	upec-136	GCA_000780695.1	UPEC	95		
Escherichia coli	upec-141	GCA_000780715.1	UPEC	NA		
Escherichia coli	upec-140	GCA_000780735.1	UPEC	127		
Escherichia coli	upec-139	GCA_000780755.1	UPEC	95		
Escherichia coli	upec-138	GCA_000780775.1	UPEC	127		
Escherichia coli	upec-135	GCA_000780795.1	UPEC	127		
Escherichia coli	upec-134	GCA_000780815.1	UPEC	131		
Escherichia coli	upec-133	GCA_000780835.1	UPEC	38		
Escherichia coli	upec-132	GCA_000780845.1	UPEC	393		
Escherichia coli	upec-131	GCA_000780875.1	UPEC	95		
Escherichia coli	upec-130	GCA_000780895.1	UPEC	101		
Escherichia coli	upec-128	GCA_000780915.1	UPEC	135		
Escherichia coli	upec-129	GCA_000780925.1	UPEC	95		
Escherichia coli	upec-127	GCA_000780965.1	UPEC	10		
Escherichia coli	upec-126	GCA_000780995.1	UPEC	70		
Escherichia coli	upec-125	GCA_000781005.1	UPEC	131		
Escherichia coli	upec-124	GCA_000781035.1	UPEC	95		
Escherichia coli	upec-123	GCA_000781045.1	UPEC	127		
Escherichia coli	upec-121	GCA_000781075.1	UPEC	297		
Escherichia coli	upec-120	GCA_000781095.1	UPEC	95		
Escherichia coli	upec-118	GCA_000781115.1	UPEC	210		
Escherichia coli	upec-119	GCA_000781145.1	UPEC	210		
Escherichia coli	upec-117	GCA_000781175.1	UPEC	73		
Escherichia coli	upec-116	GCA_000781195.1	UPEC	681		
Escherichia coli	upec-115	GCA_000781215.1	UPEC	390		
Escherichia coli	upec-114	GCA_000781235.1	UPEC	69		
Escherichia coli	upec-113	GCA_000781255.1	UPEC	1072		
Escherichia coli	upec-111	GCA_000781275.1	UPEC	2970		
Escherichia coli	upec-112	GCA_000781295.1	UPEC	646		
Escherichia coli	upec-110	GCA_000781315.1	UPEC	131		
Escherichia coli	upec-108	GCA_000781335.1	UPEC	131		
Escherichia coli	upec-109	GCA_000781355.1	UPEC	73		
Escherichia coli	upec-107	GCA_000781375.1	UPEC	636		
Escherichia coli	upec-106	GCA_000781385.1	UPEC	95		
Escherichia coli	upec-105	GCA_000781415.1	UPEC	70		
Escherichia coli	upec-104	GCA_000781435.1	UPEC	129		
Escherichia coli	upec-103	GCA_000781455.1	UPEC	3018		
Escherichia coli	blood-10-0913	GCA_000781485.1	ExPEC	131		
Escherichia coli	blood-10-0687	GCA_000781555.1	ExPEC	95		
Escherichia coli	blood-10-0686	GCA_000781575.1	ExPEC	95		
Escherichia coli	blood-10-0682	GCA_000781595.1	ExPEC	NA		
Escherichia coli	blood-10-0684	GCA_000781605.1	ExPEC	69		
Escherichia coli	blood-10-0675	GCA_000781635.1	ExPEC	69		
Escherichia coli	blood-10-0554	GCA_000781655.1	ExPEC	405		
Escherichia coli	blood-10-0541	GCA_000781675.1	ExPEC	405		
Escherichia coli	blood-10-0223	GCA_000781695.1	ExPEC	131		
Escherichia coli	blood-10-0183	GCA_000781715.1	ExPEC	38		
Escherichia coli	blood-10-0065	GCA_000781735.1	ExPEC	101		
Escherichia coli	blood-10-0040	GCA_000781755.1	ExPEC	101		
Escherichia coli	blood-10-0038	GCA_000781775.1	ExPEC	101		
Escherichia coli	blood-09-1342	GCA_000781805.1	ExPEC	393		
Escherichia coli	blood-09-1545	GCA_000781835.1	ExPEC	1193		
Escherichia coli	blood-09-1294	GCA_000781895.1	ExPEC	69		
Escherichia coli	blood-09-1290	GCA_000781915.1	ExPEC	10		
Escherichia coli	blood-09-1229	GCA_000781935.1	ExPEC	393		
Escherichia coli	blood-09-0949	GCA_000781955.1	ExPEC	167		
Escherichia coli	blood-09-0829	GCA_000781965.1	ExPEC	141		
Escherichia coli	blood-09-0856	GCA_000781995.1	ExPEC	141		
Escherichia coli	blood-09-0768	GCA_000782015.1	ExPEC	212		
Escherichia coli	blood-09-0767	GCA_000782035.1	ExPEC	420		
Escherichia coli	blood-09-0751	GCA_000782055.1	ExPEC	95		
Escherichia coli	blood-09-0718	GCA_000782075.1	ExPEC	2172		
Escherichia coli	blood-09-0626	GCA_000782095.1	ExPEC	617		
Escherichia coli	blood-09-0712	GCA_000782115.1	ExPEC	2172		
Escherichia coli	blood-09-0558	GCA_000782135.1	ExPEC	131		
Escherichia coli	blood-09-0464	GCA_000782155.1	ExPEC	405		
Escherichia coli	blood-09-0450	GCA_000782175.1	ExPEC	131		
Escherichia coli	blood-09-0428	GCA_000782195.1	ExPEC	131		
Escherichia coli	blood-09-0409	GCA_000782215.1	ExPEC	538		
Escherichia coli	blood-09-0389	GCA_000782235.1	ExPEC	538		
Escherichia coli	blood-09-0341	GCA_000782255.1	ExPEC	131		
Escherichia coli	blood-09-0249	GCA_000782275.1	ExPEC	NA		
Escherichia coli	blood-09-0074	GCA_000782295.1	ExPEC	131		
Escherichia coli	blood-09-0012	GCA_000782335.1	ExPEC	62		
Escherichia coli	blood-08-1636	GCA_000782375.1	ExPEC	393		

Escherichia coli	blood-08-1562	GCA_000782435.1	ExPEC	393		
Escherichia coli	blood-08-1493	GCA_000782455.1	ExPEC	393		
Escherichia coli	blood-08-1487	GCA_000782475.1	ExPEC	393		
Escherichia coli	blood-08-1463	GCA_000782495.1	ExPEC	131		
Escherichia coli	blood-08-1462	GCA_000782515.1	ExPEC	131		
Escherichia coli	blood-08-1447	GCA_000782535.1	ExPEC	131		
Escherichia coli	blood-08-1420	GCA_000782555.1	ExPEC	88		
Escherichia coli	blood-08-1415	GCA_000782575.1	ExPEC	393		
Escherichia coli	blood-08-1414	GCA_000782595.1	ExPEC	88		
Escherichia coli	blood-08-1318	GCA_000782615.1	ExPEC	131		
Escherichia coli	blood-08-1203	GCA_000782635.1	ExPEC	404		
Escherichia coli	blood-08-0997	GCA_000782655.1	ExPEC	404		
Escherichia coli	blood-08-0921	GCA_000782675.1	ExPEC	131		
Escherichia coli	blood-08-0654	GCA_000782695.1	ExPEC	95		
Escherichia coli	blood-08-0632	GCA_000782715.1	ExPEC	131		
Escherichia coli	blood-08-0493	GCA_000782735.1	ExPEC	95		
Escherichia coli	blood-08-0379	GCA_000782755.1	ExPEC	73		
Escherichia coli	blood-08-0215	GCA_000782775.1	ExPEC	73		
Escherichia coli	blood-08-0094	GCA_000782795.1	ExPEC	46		
Escherichia coli	blood-08-0081	GCA_000782815.1	ExPEC	69		
Escherichia coli	upec-64	GCA_000785235.1	UPEC	NA		
Escherichia coli	upec-66	GCA_000785255.1	UPEC	393		
Escherichia coli	upec-203	GCA_000785325.1	UPEC	58		
Escherichia coli	upec-10	GCA_000785355.1	UPEC	73		
Escherichia coli	100414	GCA_001265155.1	EPEC	NA		
Escherichia coli	100290	GCA_001265165.1	EPEC	236		
Escherichia coli	100343	GCA_001265175.1	EPEC	140		
Escherichia coli	102550	GCA_001265185.1	EPEC	555		
Escherichia coli	103385	GCA_001265235.1	EPEC	NA		
Escherichia coli	103573	GCA_001265255.1	EPEC	140		
Escherichia coli	203741	GCA_001265265.1	EPEC	23		
Escherichia coli	300059	GCA_001265295.1	EPEC	NA		
Escherichia coli	300262	GCA_001265315.1	EPEC	NA		
Escherichia coli	302053	GCA_001265345.1	EPEC	NA		
Escherichia coli	302048	GCA_001265355.1	EPEC			
Escherichia coli	302275	GCA_001265395.1	EPEC	NA		
Escherichia coli	401031	GCA_001265415.1	EPEC	18		
Escherichia coli	400791	GCA_001265435.1	EPEC	140		
Escherichia coli	302662	GCA_001265455.1	EPEC	140		
Escherichia coli	401140	GCA_001265465.1	EPEC	433		
Escherichia coli	401150	GCA_001265495.1	EPEC	140		
Escherichia coli	401264	GCA_001265515.1	EPEC	NA		
Escherichia coli	401954	GCA_001265535.1	EPEC	118		
Escherichia coli	402290	GCA_001265545.1	EPEC	140		
Escherichia coli	702324	GCA_001265575.1	EPEC	228		
Escherichia coli	100329	GCA_001265615.1	EPEC	169		
Escherichia coli	100854	GCA_001265625.1	EPEC	171		
Escherichia coli	102536	GCA_001265655.1	EPEC	23		
Escherichia coli	102598	GCA_001265675.1	EPEC	236		
Escherichia coli	102929	GCA_001265685.1	EPEC	535		
Escherichia coli	103578	GCA_001265715.1	EPEC	118		
Escherichia coli	200146	GCA_001265735.1	EPEC	140		
Escherichia coli	300075	GCA_001265755.1	EPEC	NA		
Escherichia coli	300214	GCA_001265765.1	EPEC	NA		
Escherichia coli	300231	GCA_001265795.1	EPEC	23		
Escherichia coli	302150	GCA_001265815.1	EPEC	NA		
Escherichia coli	302687	GCA_001265835.1	EPEC	140		
Escherichia coli	302909	GCA_001265845.1	EPEC	NA		
Escherichia coli	303145	GCA_001265875.1	EPEC	NA		
Escherichia coli	400738	GCA_001265885.1	EPEC	23		
Escherichia coli	401588	GCA_001265925.1	EPEC	NA		
Escherichia coli	401817	GCA_001265935.1	EPEC	NA		
Escherichia coli	402310	GCA_001265975.1	EPEC	NA		
Escherichia coli	402804	GCA_001265995.1	EPEC	118		
Escherichia coli	702423	GCA_001266005.1	EPEC	140		
Escherichia coli	100100	GCA_001266015.1	EPEC	78		
Escherichia coli	702626	GCA_001266025.1	EPEC	140		
Escherichia coli	100175	GCA_001266075.1	EPEC	254		
Escherichia coli	102132	GCA_001266095.1	EPEC	78		
Escherichia coli	102535	GCA_001266115.1	EPEC	NA		
Escherichia coli	103338	GCA_001266125.1	EPEC	933		
Escherichia coli	103447	GCA_001266155.1	EPEC	555		
Escherichia coli	200077	GCA_001266175.1	EPEC	20		
Escherichia coli	300469	GCA_001266195.1	EPEC	228		
Escherichia coli	300847	GCA_001266215.1	EPEC	NA		
Escherichia coli	302137	GCA_001266225.1	EPEC	378		
Escherichia coli	302312	GCA_001266255.1	EPEC	NA		
Escherichia coli	303139	GCA_001266275.1	EPEC	555		
Escherichia coli	303341	GCA_001266295.1	EPEC	140		
Escherichia coli	303301	GCA_001266305.1	EPEC	NA		
Escherichia coli	400929	GCA_001266335.1	EPEC	236		
Escherichia coli	401195	GCA_001266355.1	EPEC	18		
Escherichia coli	401675	GCA_001266375.1	EPEC	NA		
Escherichia coli	402559	GCA_001266385.1	EPEC	8		
Escherichia coli	402981	GCA_001266405.1	EPEC	23		
Escherichia coli	403341	GCA_001266435.1	EPEC	140		
Escherichia coli	700283	GCA_001266445.1	EPEC	562		
Escherichia coli	403116	GCA_001267335.1	EPEC	NA		
Escherichia coli	303289	GCA_001267345.1	EPEC	NA		
Escherichia coli	401091	GCA_001267355.1	EPEC	140		
Escherichia coli	703450	GCA_001506145.1	EPEC	NA		
Escherichia coli	EPEC11	GCA_001605745.1	EPEC	NA		

Table S4: Based on (Wurpel, et al. 2013) we extracted reference sequences used to assign monophyletic usher groups as shown in figure 1 and 2.

E. coli strain	CS1-like	CS1-CFA/I	Mat	Yhc	Type 1	F1C/S	F9	Ycb	Auf	Sfm	LPF-like	LPF	Ush	Yra	CS12	Yad	Type 3-like	Yeh	Yeh-like	F17-like	Yfc	P	P2	Pix	Yqi	Yqi-like	Ybg	EFER_1138
**E24377A**	A7ZRX9	A7ZGY2	A7ZI25					A7ZK44				A7ZTT7		A7ZS40												A7ZRS7	A7ZJ98	
**K-12 MG1655**				P45420	P30130					P77468																		
**UTI89**						Q1RDG8			Q1R5K1							Q1RG51		Q1R9W3		Q1R2V6	Q1R975	Q1R2W8			Q1R6T7			
**O127:H6 E2348/69**							B7URN8				B7ULA7		B7UIJ5															
**536**															Q0TB94									Q0TDM6				
**O157:H7 Sakai**																	Q7AFL4		Q8XA56									
**CFT073**																							Q8FAQ3					

Table S5: Predictions for the stalk/adhesin domain proteins of the respective operons with the structure online prediction tool phyre2 (Kelley and Sternberg 2009).

organism	Accession usher	Accession pilin subunit	fimbriae location	Phyre2 bes	observation	confidence	% identity	Length	Sequence
Citrobacter freundii CFNIH1	A0A023VEU2	A0A023VD70	1	fimF	1 IG domain	100	23	191	MKMKKSAVFFALLSAYSFNVLAAEMNAGTIH
Citrobacter freundii CFNIH1	A0A023VEU2	A0A023VDU5	2	lpfD	2 IG domains	100	22	380	MRHIFAVVLTSVLFLLPASVQAAITLLTNCFGA
Citrobacter koseri (strain ATCC BAA-895 / CDC 4225-83 / SGSC4696)	A8AFF4	A8AFF6	1	fimF	1 IG domain	99.9	25	191	MNARGHIISGALLALASTGTFAAELNAGTIHF
Citrobacter koseri (strain ATCC BAA-895 / CDC 4225-83 / SGSC4696)	A8AFF4	A8AFF2	2	lpfD	2 IG domains	100	22	365	MASATAQAATILRDCFTAPQYYSVTFSNEINA
Citrobacter koseri (strain ATCC BAA-895 / CDC 4225-83 / SGSC4696)	A8APL5	A8APL3	1	fimA	1 IG domain	99.9	35	172	MKKIALISAILSLSVFQAANAADGVISFTGSITG
Citrobacter koseri (strain ATCC BAA-895 / CDC 4225-83 / SGSC4696)	A8APL5	A8APL6	2	cupB6	2 IG domains	100	13	346	MPLIWRERKMYRILISLIFTSLGLFSHTALAWN
Cronobacter sakazakii (strain ATCC BAA-894) (Enterobacter sakazakii)	A7ME55	A7ME57	1	fimF	1 IG domain	99.9	30	202	MRGHFIIADIYSQKKWFWINNQGEDMKKKL
Cronobacter sakazakii (strain ATCC BAA-894) (Enterobacter sakazakii)	A7ME55	A7MFZ9	2	lpfD	2 IG domains	100	30	342	MLRKLSGVIVVCAALKTPHLQAATGFCNTTN
Enterobacter asburiae (strain LF7a)	G2S6X5	G2S6X3	1	fimF	1 IG domain	99.9	20	202	MDTSLINYMDINMNKSVLSSLLAGSIMAALSL
Enterobacter asburiae (strain LF7a)	G2S6X5	G2S6X6	2	cupB6	2 IG domains	100	22	334	MHLRRSVNTFLLPVLLVLMGAFFHPARADCT
Enterobacter lignolyticus (strain SCF1)	E3G5Q3	E3G5Q1	1	fimF	1 IG domain	100	29	192	MKRNLITGALALSSLLTVGQVLAADGTVHFR
Enterobacter lignolyticus (strain SCF1)	E3G5Q3	E3G5Q4	2	lpfD	2 IG domains	100	25	369	MKKILSGLIMLFFCYDCFAAGDGVCHNSDGS
Enterobacter lignolyticus (strain SCF1)	E3G713	E3G711	1	fimF	1 IG domain	99.9	29	188	MLQRVIIKHFNLITGFHMKNISLVSAILSLSVLC
Enterobacter lignolyticus (strain SCF1)	E3G713	E3G714	2	cupB6	2 IG domains	100	16	337	MYRLLFIIIFAAVGLFSQAALAWQCNTVTTITT
Enterobacter lignolyticus (strain SCF1)	E3GBG6	E3GBG8	1	fimA	1 IG domain	99.9	30	196	MKTLRMTYIAAGMVLAGLVSPSWATETGGV
Enterobacter lignolyticus (strain SCF1)	E3GBG6	E3GBG5*	2	cupB6	2 IG domains	100	20	354	MINRTAYRQDCLFRRVGRWLVRIALAIFFSSF
Enterobacter sp. (strain 638)	A4WBN9	A4WBN7	1	fimF	1 IG domain	99.9	24	191	MNLRKNVIAATMLAIISGSACAAETNAGTIHF
Enterobacter sp. (strain 638)	A4WBN9	A4WBP0	2	lpfD	2 IG domains	100	22	383	MLTDMKTFSRAFIGALLCLVFSASASAATTLL
Escherichia albertii KF1	W0AM47	W0AP51	1	fimF	1 IG domain	99.9	19	188	MNKKLLSLLVSGSLIAAVSTSVFAADTGVVNF
Escherichia albertii KF1	W0AM47	W0AS87	2	cupB6	2 IG domains	100	18	336	MKPGFASYIRALLTIIFLSWGVIPAVFASCNISG
Escherichia albertii KF1	W0ARU5	W0AQA2	1	fimF	1 IG domain	99.9	33	180	MEFLMKKVVFALSALALTSGSVFAADSGDGT
Escherichia albertii KF1	W0ARU5	W0AV98	2	lpfD	1.5 IG domain	100	36	305	MSNKITLFLLCGILGGFSGNALAAGGWGPCK
Escherichia albertii KF1	W0ARU5	W0AVV0	3	fimF	1 IG domain	99.8	20	178	MNRNRMFQSGLLFLALINCSDALAEKVSLGP
Escherichia coli (strain K-12 substr. MG1655)	P42915	P42913	1	fimF	1 IG domain	100	23	194	MNKVTKTAIAGLLALFAGNAAATDGEIVFDG
Escherichia coli (strain K-12 substr. MG1655)	P42915	P43319	2	lpfD	2 IG domains	100	22	363	MKRAPLITGLLLISTSCAYASSGGCGADSTSGA
Escherichia coli B7A	B3H9Y8	B3H9Y6	1	fimF	1 IG domain	100	26	190	MKRNIIGGAFTLASLMLAGHALAEDGVVHFV
Escherichia coli B7A	B3H9Y8	B3H9Y9	2	lpfD	2 IG domains	100	30	357	MNKYIKQWCFAVFMLSLSSVALAAPKGICTP
Escherichia coli O127:H6 (strain E2348/69 / EPEC)	B7UIJ5	B7UIJ4	1	fimF	1 IG domain	99.9	19	188	MNKKLLSLLVSGSFIAAVSTSVFAADTGVVNF
Escherichia coli O127:H6 (strain E2348/69 / EPEC)	B7UIJ5	B7UIJ6	2	cupB6	2 IG domains	100	17	336	MKPGFASYIRALLTIIFLSWGVIPAVFASCNISG
Escherichia coli O127:H6 (strain E2348/69 / EPEC)	B7ULA7	B7ULA9	1	fimF	1 IG domain	100	30	174	MKKVIFALSALALTSGSVFAADSGDGTVKFTG
Escherichia coli O127:H6 (strain E2348/69 / EPEC)	B7ULA7	B7ULA6	2	lpfD	2 IG domains	100	36	351	MIKILKLFVFWSILFTANNVMAADWGPCTST
Escherichia coli O127:H6 (strain E2348/69 / EPEC)	B7ULA7	B7ULA5	3	fimF	1 IG domain	99.9	24	176	MWRNSIRCLLPFLALAYFPGAEAKISLGEAGK
Escherichia coli O139:H28 (strain E24377A / ETEC)	A7ZS40	A7ZS38	1	fimF	1 IG domain	99.9	21	194	MNKVTKTAIVGLLALFAGNAAATDGEIVFDG
Escherichia coli O139:H28 (strain E24377A / ETEC)	A7ZS40	A7ZS41	2	lpfD	2 IG domains	100	21	363	MKRAPLITGLLLISTSCAYASSGGCGADSTSGA
Escherichia coli O139:H28 (strain E24377A / ETEC)	A7ZTT7	A7ZTT9	1	fimF	1 IG domain	100	26	190	MKRNIIGGAFTLASLMLAGHALAEDGVVHFV
Escherichia coli O139:H28 (strain E24377A / ETEC)	A7ZTT7	A7ZTT6	2	lpfD	2 IG domains	100	30	357	MNKYIKQWCFAVFMLSLSSVALAAPKGICTP
Escherichia coli O150:H5 (strain SE15)	D2NBW3	D2NBW1	1	fimF	1 IG domain	99.9	21	188	MNKKLLSLLVSGSLIAVVSTSVFATDTGVVNF
Escherichia coli O150:H5 (strain SE15)	D2NBW3	D2NBW4	2	cupB6	2 IG domains	100	18	336	MKPGFVSYIRTLLTIIFLSWGVIPEVFAGCNISG
Escherichia coli O150:H5 (strain SE15)	D2NFP8	c(4372031-4372600)**	1	fimF	1 IG domain	97	17	189	MNKKLLAMGLLLSMTAGSAMAAANSGTTIG
Escherichia coli O157:H7 (strain Sakai)	Q8XAB2	Q8XAB4	1	fimF	1 IG domain	99.9	21	194	MNKVTKTAIAGLLALFAGNAAATDGEIVFDG
Escherichia coli O157:H7 (strain Sakai)	Q8XAB2	Q8XAB1	2	truncated					
Escherichia coli O157:H7 (strain Sakai)	Q8XAZ9	Q8XAZ0	1	fimF	1 IG domain	99.9	31	200	MKPNMIVGALALTSVFMAGHLQAADGTVH
Escherichia coli O157:H7 (strain Sakai)	Q8XAZ9	Q8XB01	2	lpfD	2 IG domains	100	22	356	MKKILSGLILLLCCPYGFAANGDGATHMSNLS
Escherichia coli O157:H7 (strain Sakai)	Q8XAZ9	Q8XB02	3	lpfD	2 IG domains	100	24	360	MKKWHYIFCIILFHLGLPCGYAANDGTCATRG
Escherichia coli O78:H11 (strain H10407 / ETEC)	E3PK56	E3PK54	1	fimF	1 IG domain	99.9	23	194	MNKVTKTAIAGLLALFAGNAAATDGEIVFDG
Escherichia coli O78:H11 (strain H10407 / ETEC)	E3PK56	E3PK57	2	lpfD	2 IG domains	100	22	363	MKRAPLITGLLLISTSCAYASSGGCGADSTSGA
Escherichia coli O9:H4 (strain HS)	A8A4W0	A8A4V8	1	fimF	1 IG domain	99.9	22	194	MNKGTKIAIAGLLALFAGNAAATDGEIVFDGE
Escherichia coli O9:H4 (strain HS)	A8A4W0	A8A4W1	2	lpfD	2 IG domains	100	22	363	MKRAPLITGLLLISTSCAYASSGGCGADSTSGA
Klebsiella oxytoca (*K. michiganensis*) (strain ATCC 8724 / DSM 4798 / JCM 20051 / NBRCOS 3318 / NRRL B-19	G8WAR5	G8WAR7	1	fimF	1 IG domain	99.9	31	198	MKTQFRIMAMFVGALVSASALSSPSQPARSA
Klebsiella oxytoca (*K. michiganensis*) (strain ATCC 8724 / DSM 4798 / JCM 20051 / NBRCOS 3318 / NRRL B-19	G8WAR5	G8WAR4	2	cupB6	2 IG domains	100	16	364	MRIVSFLFLLMAGVYSHSGWAETCRGDIGQ
Klebsiella pneumoniae (strain 342)	B5XQQ9	B5XQQ7	1	fimA	1 IG domain	99.9	22	187	MKKKGTLLVVGSLLALSLATVTTARAAGTLNF
Klebsiella pneumoniae (strain 342)	B5XQQ9	B5XQR0	2	cupB6	2 IG domains	100	16	334	MSFLTLLRRATLTLLLALLPPASGWACTPVFVR
Klebsiella pneumoniae (strain 342)	B5XVG0	B5XVF7	1	fimF	1 IG domain	99.9	32	197	MKTQLRIMAILMGALVSGAAISGNTQPASSA
Klebsiella pneumoniae (strain 342)	B5XVG0	B5XVG1	2	cupB6	2 IG domains	100	17	364	MKSVFRLLPVLFLLALSGYLPGVRAETCRANIG
Klebsiella pneumoniae 30684/NJST258_2	W8UDN2	W8UVV7	1	fimF	1 IG domain	99.9	30	193	MKKQPRFITILIGTLLSGVALQATAANTINPDS
Klebsiella pneumoniae 30684/NJST258_2	W8UDN2	W8UG85	2	cupB6	2 IG domains	100	18	364	MKSVFRLLPFLFLLGLSAYLPGVRAETCRANIG

Klebsiella pneumoniae 30684/NJST258_2	W8UKG8	W8UI31	1	fimA	1 IG domain	99.9	22	189	MSMKEKGTLLVVGSLLALSLATVTTARAAGTL
Klebsiella pneumoniae 30684/NJST258_2	W8UKG8	W8V048	2	cupB6	2 IG domains	100	18	334	MSFLTLLRRVAMTLLFILLPAASGWACTPVFV
Klebsiella pneumoniae subsp. pneumoniae (strain ATCC 700721 / MGH 78578)	A6T931	A6T933	1	fimA	1 IG domain	99.9	22	187	MKEKGTLLVVGSLLALSLATVTTARAAGTLNF
Klebsiella pneumoniae subsp. pneumoniae (strain ATCC 700721 / MGH 78578)	A6T931	A6T930	2	cupB6	2 IG domains	100	17	334	MSFLTLLRRVALTLLFTLLPAASGWACTPVFV
Klebsiella pneumoniae subsp. pneumoniae (strain ATCC 700721 / MGH 78578)	A6TCS3	A6TCS5	1	fimF	1 IG domain	99.9	30	193	MKKQPRFITILIGALLSGVALQATAANTINPDS
Klebsiella pneumoniae subsp. pneumoniae (strain ATCC 700721 / MGH 78578)	A6TCS3	A6TCS2	2	cupB6	2 IG domains	100	17	364	MKSVFRLLPFLFLLGLSAYLPGVRAETCRANIG
Klebsiella pneumoniae subsp. pneumoniae PittNDM01	A0A075VC40	A0A075V8V7	1	fimA	1 IG domain	99.9	21	187	MKEKGTLLVVGSLLALSLATVTTARAAGTLNF
Klebsiella pneumoniae subsp. pneumoniae PittNDM01	A0A075VC40	A0A075VGC7	2	cupB6	2 IG domains	100	18	334	MSFLTLLRRVAMTLLFILLPAASGWACTPVFV
Klebsiella pneumoniae subsp. pneumoniae PittNDM01	A0A075VGG1	A0A075VR62	1	fimF	1 IG domain	99.9	30	193	MKKQPRFITILIGALLSGVALQATAANTINPDS
Klebsiella pneumoniae subsp. pneumoniae PittNDM01	A0A075VGG1	A0A075VJ98	2	cupB6	2 IG domains	100	19	364	MKSVFRLLPFLFLLGLSAYLPGVRAETCRANIG
Klebsiella variicola (strain At-22)	D3RFR9	D3RFR7	1	fimA	1 IG domain	99.9	21	187	MKEKGTLLVVGSLLAFSLATVTPARAAGTLNF
Klebsiella variicola (strain At-22)	D3RFR9	D3RFS0	2	cupB6	2 IG domains	100	18	334	MSFLTLLRRATLTLLLALLPPASGWACTPVFV
Klebsiella variicola (strain At-22)	D3RJK6	D3RJK4	1	fimF	1 IG domain	99.9	32	197	MKTQLRIMAILMGALVSGAAISGNTQPARST
Klebsiella variicola (strain At-22)	D3RJK6	D3RJK7	2	cupB6	2 IG domains	100	17	364	MKSVFRLLPVLFLLALSGYLPGVRAETCRANIG
Salmonella bongori N268-08	S5N1N5	S5NE28	1	fimF	1 IG domain	99.9	33	174	MKKVIFALSALAVVSTSAFAAESGDGTIKFTGE
Salmonella bongori N268-08	S5N1N5	S5NKU9	2	lpfD	2 IG domains	100	43	354	MLKKLMTFAGLMGGSVLFSGQAAAADWGP
Salmonella bongori N268-08	S5N1N5	S5MW79	3	fimF	1 IG domain	99.9	21	175	MKTLHALMPACLLLTASVMAAPSNIGSAGDI
Salmonella bongori N268-08	S5N468	S5NN90	1	fimF	1 IG domain	99.9	34	170	MKKTMLSACFMLAAANAMAADGTIHFTGSI
Salmonella bongori N268-08	S5N468	S5NGE2	2	cupB6	2 IG domains	100	21	317	MKGLLKSGCLLLALWLVAFGAQASCQFANG
Salmonella bongori N268-08	S5NLK0	S5N2J6	1	fimF	1 IG domain	99.9	31	191	MKPNLIVSALVLSTSMLVGQAMASDGTVHF
Salmonella bongori N268-08	S5NLK0	S5MWU1	2	lpfD	2 IG domains	100	27	334	MVCFYHTVFAADTYCEADGGVHHSILVFTGT
Salmonella typhimurium (strain LT2 / SGSC1412 / ATCC 700720)	P43662	P43660	1	fimF	1 IG domain	100	33	178	MEFLMKKVVFALSALAVVSTSAFAAESGDGT
Salmonella typhimurium (strain LT2 / SGSC1412 / ATCC 700720)	P43662	P43663	2	lpfD	2 IG domains	100	46	359	MLKKLIMFTGLLGGSVLFSGQALAAADFGPC
Salmonella typhimurium (strain LT2 / SGSC1412 / ATCC 700720)	P43662	P43664	3	fimF	1 IG domain	99.9	20	175	MKNLHALMPACLLLTASAMAAPSNIGSAGDI
Salmonella typhimurium (strain SL1344)	E1WD67	E1WD69	1	fimF	1 IG domain	100	33	178	MEFLMKKVVFALSALAVVSTSAFAAESGDGT
Salmonella typhimurium (strain SL1344)	E1WD67	E1WD66	2	truncated					
Salmonella typhimurium (strain SL1344)	E1WD67	E1WD65	3	fimF	1 IG domain	99.9	20	175	MKNLHALMPACLLLTASAMAAPSNIGSAGDI
Shigella flexneri 2a str. 2457T	Q83PM2	Q83J02	2	truncated					
Shigella sonnei (strain Ss046)	Q3YX98	Q3YX96	2	lpfD	2 IG domains	100	21	317	MSGNIGANPFTSATLSSTNWQYACSCSAGKA
Yersinia enterocolitica LC20	W8U5A0	W8UMD5	1	fimF	1 IG domain	99.9	26	177	MNKGFIAISLLALSSSAFAIDGTIQFSGAFTDSP
Yersinia enterocolitica LC20	W8U5A0	W8UHH1	2	cupB6	2 IG domains	100	18	347	MHFKLRKIMKRMILPLFISVASIAPAYSINSNL
Yersinia enterocolitica LC20	W8U5A0	W8UMC9	3	fimF	1 IG domain	99.9	30	170	MMTLLWRGLFLALLIAAPVVQAADVAFNITG
Yersinia enterocolitica LC20	W8V9V3	W8U601	1	fimF	1 IG domain	99.9	19	171	MNMKNKLIVTSLLAASAFASVANAGVFNITG
Yersinia enterocolitica LC20	W8V9V3	W8UN19	2	truncated					
1.5 IG domain: probably two, but second only partially predicted									
*The annotated sequence was truncated (240aa), so the sequence was extended to include the upstream start codon (354aa)									
**Sequence was not annotated originally, manually enterred the sequence									
